# Overexpression of OsMYB305 in Rice Enhances the Nitrogen Uptake Under Low-Nitrogen Condition

**DOI:** 10.3389/fpls.2020.00369

**Published:** 2020-04-15

**Authors:** Dujun Wang, Tangqian Xu, Zikui Yin, Wenjuan Wu, Haoting Geng, Long Li, Meng Yang, Hongmei Cai, Xingming Lian

**Affiliations:** National Key Laboratory of Crop Genetic Improvement and National Center of Plant Gene Research, Huazhong Agricultural University, Wuhan, China

**Keywords:** OsMYB305, transcription factor, rice, nitrogen uptake, cellulose synthesis

## Abstract

Excessive nitrogen fertilizer application causes severe environmental degradation and drives up agricultural production costs. Thus, improving crop nitrogen use efficiency (NUE) is essential for the development of sustainable agriculture. Here, we characterized the roles of the MYB transcription factor *OsMYB305* in nitrogen uptake and assimilation in rice. *OsMYB305* encoded a transcriptional activator and its expression was induced by N deficiency in rice root. Under low-N condition, *OsMYB305* overexpression significantly increased the tiller number, shoot dry weight and total N concentration. In the roots of *OsMYB305*-OE rice lines, the expression of *OsNRT2.1*, *OsNRT2.2*, *OsNAR2.1*, and *OsNiR2* was up-regulated and ^15^NO_3_^–^ influx was significantly increased. In contrast, the expression of lignocellulose biosynthesis-related genes was repressed so that cellulose content decreased, and soluble sugar concentration increased. Certain intermediates in the glycolytic pathway and the tricarboxylic acid cycle were significantly altered and NADH-GOGAT, Pyr-K, and G6PDH were markedly elevated in the roots of *OsMYB305*-OE rice lines grown under low-N condition. Our results revealed that *OsMYB305* overexpression suppressed cellulose biosynthesis under low-nitrogen condition, thereby freeing up carbohydrate for nitrate uptake and assimilation and enhancing rice growth. OsMYB305 is a potential molecular target for increasing NUE in rice.

## Introduction

Rice is a dietary staple for ∼50% of the global population (Godfray et al., 2010). Nitrogen (N) is the most important and limiting macronutrient for rice growth and productivity. To meet increasing worldwide food demands, growers have applied nitrogen fertilizer in the attempt to maximize yields (Huang et al., 2017). The global agricultural food production doubled during the past 35 years but the quantity of N fertilizer applied to realize this gain increased by 6.9-fold (Zhang et al., 2011). Only ∼30–50% of the applied N is actually absorbed by the rice (Socolow, 1999). The balance is lost to the ambient environment in the form of ammonia volatilization, denitrification, surface runoff and leaching. These processes cause serious environmental pollution such as soil acidification, air pollution and aquatic eutrophication, and drive up agricultural production costs (Li H. et al., 2017; Ali et al., 2018). Therefore, the improvement of nitrogen use efficiency (NUE) in rice is urgently needed in order to achieve “low input but high output” (Zhang, 2007; Xu et al., 2012; Gojon, 2017; Swarbreck et al., 2019).

Urea is the principle form of N fertilizer used in agriculture practice. It is rapidly degraded into ammonium by urease in the soil (Kojima et al., 2006; Wang et al., 2012). Hence, ammonium is the major nitrogen form in flooded soil environments. Rice roots release oxygen from their aerenchyma, induce rapid nitrification in the rhizosphere, and absorb NO_3_^–^ at rates comparable to those for NH_4_^+^ (Kirk and Kronzucker, 2005; Li et al., 2008). Nitrate uptake is mediated by NRT1/NPF (low-affinity) and NRT2 (high-affinity) family transporters while ammonium absorption is regulated by ammonium/methylammonium transporters (AMTs) (Feng et al., 2011; Li et al., 2012; Yan et al., 2011). The NH_4_^+^ derived from NO_3_^–^ or directly absorbed by AMTs is assimilated into amino acids via the glutamine synthetase (GS)/glutamine-2-oxoglutarate aminotransferase (GOGAT) cycle and translocated to developing organs (Li H. et al., 2017).

Numerous studies have been conducted to improve rice NUE by manipulating the genes directly involved in N uptake and assimilation (Gojon, 2017). Constitutive expression of the barley amino transferase gene (AlaAT) in rice root dramatically elevated NUE and grain yield (Shrawat et al., 2008). *OsNR2* from *indica* rice (9311) exhibited greater NR activity than its corresponding allele in the *japonica* cultivar (Nipponbare). The introduction of *indica OsNR2* into Nipponbare increased its effective tiller number, grain yield, and NUE (Gao et al., 2019). Overexpression of *OsPTR9* (*OsNPF8.20*), *OsPTR6* (*OsNPF7.3*), and *OsNPF7.2* improved rice growth and grain yield (Fang et al., 2013, 2017; Fan et al., 2014; Wang J. et al., 2018). Introduction of *OsNRT1.1B* (*OsNPF6.5*) from *indica* cultivars into *japonica* cultivars enhanced nitrate absorption and NUE (Hu et al., 2015). *OsNRT1.1A* (*OsNPF6.3*) overexpression in rice greatly improved grain yield and N utilization and significantly shortened maturation time (Wang W. et al., 2018). Increasing OsNRT2.1 transcripts driven by the *OsNAR2.1* promoter significantly improved rice yield and NUE (Chen et al., 2016). Overexpression of *OsNAR2.1* via its native promoter also increased nitrogen uptake, rice growth and yield (Chen et al., 2017). Overexpression of the high-affinity nitrate transporter *OsNRT2.3b* increased N, Fe, and P uptake and significantly improved NUE and grain yield (Fan et al., 2016; Feng et al., 2017). In contrast, overexpression of the ammonium transporters *OsAMT1.1* and *OsAMT1.3* in rice increased ammonium absorption but also impaired growth and lowered grain yield (Hoque et al., 2006; Kumar et al., 2006; Bao et al., 2015). Overexpression of the glutamine synthetases *OsGS1.1* and *OsGS1.2* required for primary N assimilation reduced grain yield and disrupted C metabolism in rice (Cai et al., 2009).

There is a close relationship between N and carbon (C) metabolism. N assimilation requires C metabolism to provide adenosine triphosphate (ATP), reducing agents and carbon skeletons. In turn, nitrogen availability influences the distribution of assimilated carbon among organic acids and structural- and non-structural carbohydrates (Foyer et al., 2011; Li G. H. et al., 2018; Ohashi et al., 2018b; Xin et al., 2019). Several reports proposed that coordinating the C:N balance is vital for healthy and normal plant growth, development and yield formation (Nunes-Nesi et al., 2010; Sun et al., 2013; Li et al., 2016). The regulation of N and C metabolism in plants is complex. Several transcription factors (TFs) were regarded as potential targets for NUE improvement as they coordinately control the expression of genes involved in these processes. Introduction of the *ZmDof1* into rice significantly accelerated the photosynthesis and carbon flux toward N assimilation. In this way, it improved N utilization and plant growth under low-N conditions (Kurai et al., 2011). Overexpression of the Dof transcription factor *OsRDD1* facilitated the uptake and accumulation of NH_4_^+^, Na^+^, SO_4_^2–^, Cl^–^ PO_4_^3–^ and sucrose in rice (Iwamoto and Tagiri, 2016). A recent study showed that OsGRF4 directly binds the promoters of the genes participating in nitrogen assimilation and carbon fixation. An increase in OsGRF4 protein abundance markedly improved rice grain yield and NUE (Li S. et al., 2018).

Extensive transcriptome analyses have been conducted on rice to identify genome-wide changes in gene expression in response to nitrogen starvation (Lian et al., 2006; Cai et al., 2012; Yang et al., 2015; Hsieh et al., 2018; Shin et al., 2018). These studies revealed that in order to contend with nitrogen deficiency, rice must modulate the expression levels of extensive genes involved in N assimilation, C metabolism, root architecture formation, chlorophyll biosynthesis and stress response. Numerous transcription factors have been isolated that respond to low-N stress. These are putative research candidates for NUE improvement in rice (Cai et al., 2012; Yang et al., 2015; Hsieh et al., 2018).

However, the functions of these transcription factors (TFs) in nitrogen uptake and assimilation have not yet been elucidated. Here, we investigated the effects of the nitrogen deficiency-induced transcription factor *OsMYB305* on low-N adaptation in rice. We generated *OsMYB305*-overexpressing lines and compared their performances in hydroponic culture under various nitrogen levels. *OsMYB305* overexpression enhanced N uptake and assimilation, improved growth under low nitrogen conditions, reduced cellulose biosynthesis and altered carbon metabolism in rice. Our discoveries provide an empirical basis for the improvement of nitrogen uptake in rice by manipulating carbohydrate allocation between N assimilation and secondary cell wall biosynthesis.

## Materials and Methods

### Plant Materials and Growth Conditions

To generate the overexpression construct, the full-length coding sequence of *OsMYB305* was amplified from the total cDNA of rice cultivar Zhonghua11 (ZH11). The primers were designed according to the Os01g0637800 sequence from the NCBI database^1^. The PCR product was recombined with pDONR207 (Invitrogen, Carlsbad, CA, United States) to obtain the entry clones. Error-free clones were introduced into the destination vector pJC034 by LR recombination (Zhang et al., 2018). The constructs were introduced into *Agrobacterium tumefaciens* strain EHA105 and transferred into cv. Zhonghua11 as previously described (Hiei et al., 1994).

To generate *OsMYB305*-CRISPR vector, we designed the DNA spacer using an online tools^2^ (Lei et al., 2014). The amplified PCR product including U3 promoter, spacer of *OsMYB305* and sgRNA (small guide RNA) were cloned into vector PJE45/pH-Ubi-cas9-7 (Miao et al., 2013).

For the hydroponic culture, WT and *OsMYB305*-overexpressing rice seeds were germinated on filter paper soaked with distilled water for 3 days at 37°C and then sown in sand. Seedlings at the four-leaf stage were transplanted into a hydroponic culture container in the greenhouse. The nutrient solutions were prepared as previously described (Yoshida et al., 1976) and refreshed every 3 days. NH_4_NO_3_ was supplied as a nitrogen source at 0.288 mM (low nitrogen, LN), 0.72 mM (moderate nitrogen, MN) and 1.44 mM (high nitrogen, HN).

### Phylogenetic Analysis

All sequences were obtained from the NCBI protein database by blasting the full-length OsMYB305 protein sequence. The defined species included *Oryza sativa*, *Zea mays*, *Arabidopsis thaliana*, *Nicotiana tabacum*, and *Antirrhinum majus*. Closely related protein sequences were aligned by ClustalW integrated into the MEGA-X program. The aligned sequences were then used to produce phylogenetic trees in MEGA-X (Kumar et al., 2018) by the neighbor-joining method. One thousand bootstrap replications per tree were produced to test for robustness.

### Subcellular Localization

To determine the subcellular localization of OsMYB305, a full-length *OsMYB305* coding sequence was cloned into the pH7WGF2 vector to produce a GFP-OsMYB305 fusion construct driven by a cauliflower mosaic virus 35S promoter (35S:GFP:OsMYB305). OsbZIP46 was previously localized to the nucleus (Tang et al., 2012) and cloned into the pB7WGR2 vector to produce the RFP-OsMYB305 fusion construct as a nuclear marker. The two aforementioned constructs were co-transformed into rice protoplasts prepared from etiolated shoots by polyethylene glycol treatment (Tang et al., 2012). The fluorescence signal was observed under a confocal microscope (FV1000; Olympus, Tokyo, Japan) 16 h after transformation.

### Luciferase Assays

An effector plasmid was constructed by fusing OsMYB305 with a GAL4 DNA-binding domain (GAL4DB) under the control of a CaMV 35S promoter (GAL4DB-MYB305). An empty vector (none) was used as a control. The reporter plasmid contained firefly luciferase gene driven by five copies of the GAL4 binding site (5 × GAL4) or the CaMV 35S promoter + 5 × GAL4 (Hao et al., 2011). The *Renilla* luciferase gene (*R-luc*) driven by the 35S promoter was simultaneously co-transfected as an internal control. The luciferase assay (E1910; Promega, Madison, WI, United States) was conducted according to the manufacturer’s instructions. Fluorescence was detected with a microplate spectrophotometer (SPARK; Tecan GmbH, Grödig, Austria).

### Gene Expression Analysis

Transgenic and wild type roots and leaves were harvested at tillering stage from three biological replicates under the LN, MN, and HN conditions. Total RNA was extracted with Trizol reagent (Invitrogen, Carlsbad, CA, United States) according to the manufacturer’s instructions. First-strand cDNAs were synthesized with M-MLV reverse transcriptase (Invitrogen, Carlsbad, CA, United States) from DNase I-treated total RNA. The RT-qPCR reaction was run in an optical 384-well plate containing SYBR Premix Ex Taq^TM^ (TaKaRa Bio Inc., Shiga, Japan) and a QuantStudio 6 Flex PCR system (Applied Biosystems, Foster City, CA, United States). All gene-specific primers for the RT-qPCR were designed according to the cDNA sequences and are listed in Supplementary Table S1. The specific primer for the rice *UBI* gene (XM_015783851.1) was used as an internal control. The relative gene expression level was evaluated by the 2^–ΔΔCt^ method (Livak and Schmittgen, 2001).

### Total N Concentration Assays

Rice shoots and roots were harvested, dried at 80°C for 48 h, and ground into powder. Then 0.2 g powder was digested with 5 mL concentrated sulfuric acid at 376°C for 30 min. Then 1 mL hydrogen peroxide was added, and the samples were maintained at 376°C for 10 min. This process was repeated thrice. The samples were then stored at 376°C for 90 min. After cooling, the sample volumes were made up to 100 mL using deionized water. Then, the samples were reacted with sodium salicylate and sodium dichloroisocyanurate and the absorbance was measured at 660 nm in a SmartChem 200 (AMS Alliance, Frépillon, France). The device was calibrated with an ammonium sulfate standard, a calibration curve was plotted, and the total N concentrations were interpolated from it.

### ^15^N Trace Assays

Nitrogen influx rate was assayed with ^15^N as previously described (Bao et al., 2015). Wild type and *OsMYB305*-OE rice seedlings were grown in International Rice Research Institute (IRRI) nutrient solution for 2 weeks under 0.2 mM NH_4_NO_3_ and 2 mM NH_4_NO_3_. For the ^15^NO_3_ uptake analysis, the seedlings were transferred to 0.1 mM CaSO_4_ for 1 min, IRRI nutrient solution containing either 0.2 mM or 2 mM NH_4_^15^NO_3_ (atom% 15N:15NO_3_^–^ = 98%) for 10 min, and 0.1 mM CaSO_4_ for 1 min before sampling. For the ^15^NH_4_^+^ uptake analysis, the method was the same as that described above except ^15^NH_4_NO_3_ (atom% 15N:15NH_4_^+^ = 98%) was the labeled N source instead. Four biological replicates were harvested, dried at 70°C, and ground to powder. The ^15^N concentrations were measured in an isotope mass spectrometer (Isoprime100; Elementar Analysensysteme GmbH, Langenselbold, Germany).

### Nitrate, Ammonium and Free Amino Acid Assays

Fresh rice roots and shoots were freeze-dried and ground to powder. For the nitrate and ammonium analyses, 20 mg powder was extracted with 1 mL H_2_O at 80°C for 20 min. After centrifugation (13,000 × *g*, 10 min), the nitrate and ammonium concentrations in the supernatant were measured as previously described (Sarasketa et al., 2014). For the free amino acid analysis, 100 mg powder was extracted with 1 mL of 80% (v/v) methanol at 4°C for 12 h and centrifuged (4°C, 13,000 × *g*, 10 min). The supernatant was passed through 0.45 μm cellulose acetate centrifuge tube filters and analyzed by HPLC.

### Total C, Starch, and Soluble Sugar Assays

To measure the total C concentrations, the roots and shoots of rice seedlings grown in LN and HN conditions were harvested and dried at 80°C. Then the samples were ground to powder and ∼5 mg powder was examined in an isotope mass spectrometer (Isoprime100; Elementar Analysensysteme GmbH, Langenselbold, Germany).

Soluble sugar and starch were determined according to a previously described method (Li G. H. et al., 2018). Briefly, oven-dried rice tissue samples were ground to powder and ∼100 mg powder was extracted with 5 mL of 80% (v/v) ethanol at 80°C for 30 min. This process was repeated thrice. After centrifugation (13,000 × *g*, 10 min), the supernatants were collected, and their volume was made up to 100 mL using deionized water. This solution was then used to determine the soluble sugar concentrations.

To determine the starch concentration, the solid residue remaining in the centrifuge tube was resuspended in 2 mL distilled water and boiled in a water bath for 15 min. After cooling, 2 mL of 9.2 M HClO_4_ was added and the tube was intermittently stirred for 15 min. After centrifugation (13,000 × *g*, 10 min), the supernatant was collected in a 100 mL volumetric flask. The aforementioned extraction steps were repeated by adding 2 mL of 4.6 M HClO_4_ for 15 min. The supernatants were combined, and their volume was made up to 100 mL using deionized water.

The soluble sugar and starch concentrations were determined by colorimetry with anthrone reagent in a microplate spectrophotometer (SPARK; Tecan GmbH, Grödig, Austria) at 620 nm. A standard glucose curve was plotted, and the soluble sugar and glucose concentrations were interpolated from it. The starch concentration was estimated by multiplying the glucose concentration by 0.9. The sucrose, glucose and fructose concentrations were measured with an assay kit (K-SUFRG; Megazyme International Ireland Ltd., Bray Business Park, Bray, Co. Wicklow, Ireland) according to the manufacturer’s instructions.

### Metabolite Analyses

The rice tissue metabolites were measured by LC-MS/MS according to a previously described method (Guo et al., 2014). Fresh rice roots and shoots were harvested and ground to a powder in liquid nitrogen. Then 200 mg powder was weighed into a 10 mL plastic tube to which 3 mL methanol/chloroform (7:3, v/v; −20°C) was added. The suspension was vortexed and 40 μL of 300 μM PIPES [piperazine-N,*N*’-*bis*(2-ethanesulfonic acid)] was added as the internal standard. The mixtures were intermittently vortexed for 2 h at −20°C. Polar metabolites were extracted from the methanol/chloroform phase by adding 2.4 mL water to each sample, vortexing, and centrifuging (4°C, 13,000 × *g*, 5 min). The methanol-water phase was then transferred to a new tube and the polar metabolite extraction was repeated. The extracts were then pooled and dried at room temperature by N_2_ aspiration. The dried extract was re-dissolved in 200 μL deionized water, passed through a 0.45 μm cellulose acetate centrifuge tube filter and analyzed by LC-MS/MS.

### Cell Wall Composition Analyses

Cell wall residues of wild type and transgenic plants were converted into alcohol-insoluble residues (AIR) and de-starched as previously described (Huang et al., 2015). To determine the hemicellulose content, the pretreated AIR was hydrolyzed in 2 M HCl at 100°C for 1 h and the supernatant OD was measured (540 nm) after reaction with 3,5-dinitrosalicylic acid. The residue was then washed and hydrolyzed with 72% (v/v) sulfuric acid. The cellulose content was measured by anthrone assay (Li et al., 2009). The lignin content was measured by the acetyl bromide method as previously described (Xue et al., 2019).

### Enzyme Activity Analyses

Enzyme extracts from the rice roots and shoots were prepared as previously described (Gibon et al., 2004). The activity levels of NADP-ICDH, Ala aminotransferase (AlaAT), Asp aminotransferase (AspAT), fructose-1,6-bisphosphatase (FBPase), glucokinase (GlcK), fructokinase (FruK), NADP-glucose-6-phosphate dehydrogenase (G6PDH), shikimate dehydrogenase (ShikDH), and PEP carboxylase (PEPC) were detected as previously described (Gibon et al., 2004). The NADH and NADPH fluorescence levels were monitored at 340 nm in a microplate spectrophotometer (SPARK; Tecan GmbH, Grödig, Austria). Nitrate reductase (NR), nitrite reductase (NiR), glutamine synthetase (GS), and NADP-malic enzyme (ME) were assayed as previously described (Migge et al., 1997; Jenner et al., 2001; Gibon et al., 2004).

## Results

### *OsMYB305* Is Induced by N Deficiency in Rice Root

**FIGURE 1:**
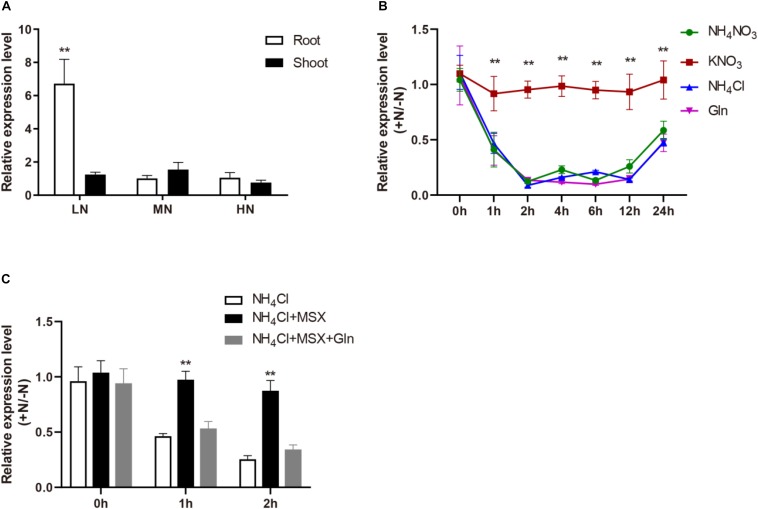
The expression pattern of *OsMYB305* responded to nitrogen. **(A)** The expression of *OsMYB305* under different concentrations of nitrogen. The rice seedlings (cv. *ZH11*) were hydroponic cultured under 0.288 mM (LN), 0.72 mM (MN), and 1.44 mM (HN) NH_4_NO_3_ treatments for 4 weeks, then the expression of *OsMYB305* in roots and shoots were measured. **(B)** The expression of *OsMYB305* responded to different N sources. The rice seedlings were pretreated with N-free medium for 7 days, then resupplied with KNO_3_ (2.88 mM), NH_4_Cl (2.88 mM), NH_4_NO_3_ (1.44 mM), Gln (1.44 mM), respectively. The samples were harvested at 0, 1, 2, 4, 6, 12, and 24 h after N resupply. The relative expression level represented the ratio of the values under N-resupplying to the values under N- deficiency. **(C)** The expression of *OsMYB305* treating with MSX. Methionine sulfoximine (MSX) was used to inhibit the activity of glutamine synthetase (GS). The rice seedlings pretreated with N-free medium for 7 days, were resupplied with NH_4_Cl (2.88 mM), NH_4_Cl (2.88 mM) + MSX (1 mM) and NH_4_Cl (2.88 mM) + MSX (1 mM) + Gln (1.44 mM). The root samples were harvested at 0, 1, and 2 h after N supply. The relative expression level was calculated by the ratio of the values under N-resupplying to the values under N-deficiency. Values were means ± SD of 3 biological replications. Statistical significance was determined using Student’s *t*-test (**P < 0.01).

We detected *OsMYB305* expression in various rice tissues using RT-PCR and real-time q-PCR. Although *OsMYB305* was preferentially expressed in the young panicles, its transcripts were also found in the roots, leaves, leaf sheaths, nodes and internodes (Supplementary Figures S1A,B). To explore the *OsMYB305* expression pattern in response to nitrogen, rice seedlings were grown for 4 wks in culture solutions containing 0.288, 0.72, and 1.44 mM NH_4_NO_3_ (LN, MN, and HN) and the *OsMYB305* expression levels in root and shoot were measured. Under LN, the *OsMYB305* expression level in the root was significantly higher (∼6×) than those for the MN and HN conditions. In contrast, the *OsMYB305* expression level in the shoot did not differ among treatments (Figure 1A).

To investigate the responses of *OsMYB305* to various nitrogen sources, rice seedlings were pretreated with N-free medium for 7 days and then resupplied with different nitrogen sources. Tissue samples were harvested at 0, 1, 2, 4, 6, 12, and 24 h after nitrogen reintroduction. At 2.88 mM KNO_3_, the *OsMYB305* expression did not markedly change compare with that under the N-deprived condition. On the other hand, when NH_4_NO_3_, NH_4_Cl, and Gln were administered as N sources, *OsMYB305* expression was significantly repressed (Figure 1B).

To establish whether *OsMYB305* expression was, in fact, repressed by NH_4_^+^ or Gln, methionine sulfoximine (MSX) was added to block Gln synthesis from NH_4_^+^ (Ohashi et al., 2018a). MSX reversed the NH_4_Cl-induced repression of *OsMYB305* expression while Gln recovered it in the presence of MSX (Figure 1C).

### *OsMYB305* Encodes a Transcriptional Activator Which Is Localized to the Nucleus

**FIGURE 2:**
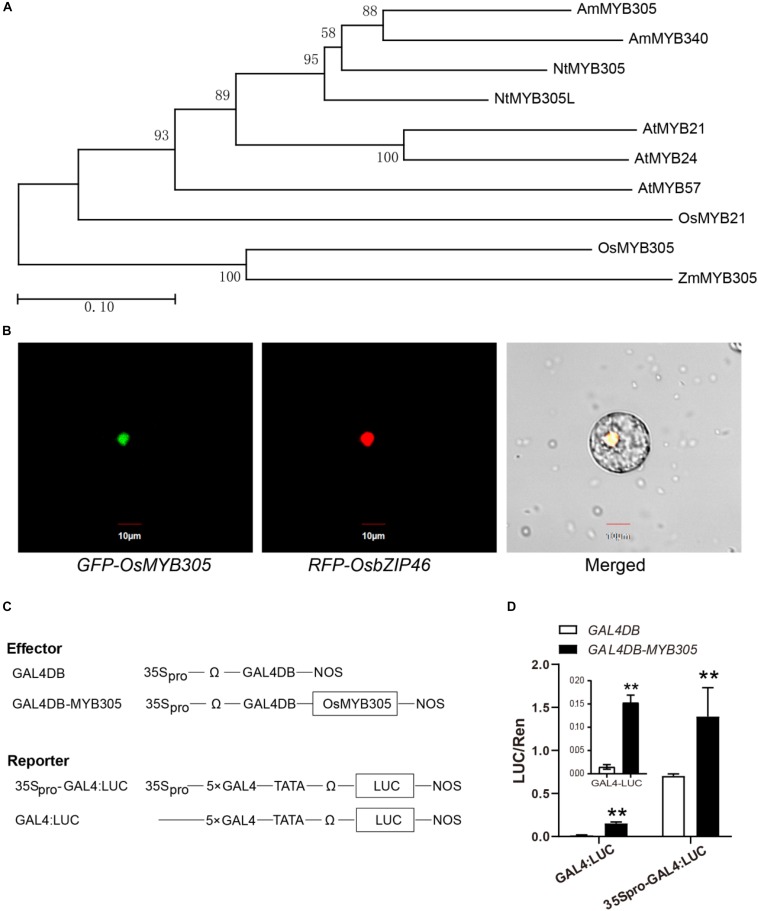
Phylogenetic analysis, subcellular localization and transcriptional activation analysis of OsMYB305. **(A)** Phylogenetic analysis of OsMYB305. The homologous proteins of OsMYB305 in the species: *Oryza sativa* (Os), *Zea mays* (Zm), *Arabidopsis thaliana* (At), *Nicotiana tabacum* (Nt), and *Antirrhinum majus* (Am), were used to construct the phylogenetic tree. The figures mean bootstrap values. The scale was for the length of branch. **(B)** Subcellular localization of OsMYB305. OsMYB305 was fused with GFP at the N terminal. The fused protein RFP-OsbZIP46 was used as the nucleus marker. **(C)** The construction of the effector and reporter used in dual-Luciferase reporter assay. **(D)** Transcriptional activation test using dual-Luciferase reporter assay. The effector and reporter vectors were co-transformed into rice protoplasts. After 16 h, the florescence of Luciferase and Renilla luciferase (internal control) were detected, and the ratios of Luc/Ren were calculated. Values were means ± SD of 3 biological replications. Statistical significance was determined using Student’s *t*-test (**P < 0.01).

A phylogenetic analysis revealed that OsMYB305 belongs to the R2R3-MYB transcription factor family, and is highly similar to ZmMYB305, NtMYB305, AmMYB305, and the three MYB21-clade proteins AtMYB21, AtMYB24, and AtMYB57 in Arabidopsis (Figure 2A). We then conducted the phylogenetic analysis using the whole R2R3-MYB family proteins in rice (Zhao and Bartley, 2014), and found that OsMYB305 has a few closely related proteins (Supplementary Figure S2). A subcellular location analysis with rice protoplast showed that the GFP-OsMYB305 fusion protein was localized to the nucleus and colocalized with the nuclear marker RFP-OsbZIP46 (Figure 2B). To test whether OsMYB305 activates transcription, a dual-luciferase reporter assay was performed on rice protoplasts. GAL4DB-MYB305 significantly activated the 5 × GAL4:LUC (> 10×) and 35Spro-5 × GAL4:LUC (∼2×) reporters (Figures 2C,D).

### *OsMYB305* Overexpression Increases Rice Growth and N Concentration Under Low-Nitrogen Condition

**FIGURE 3:**
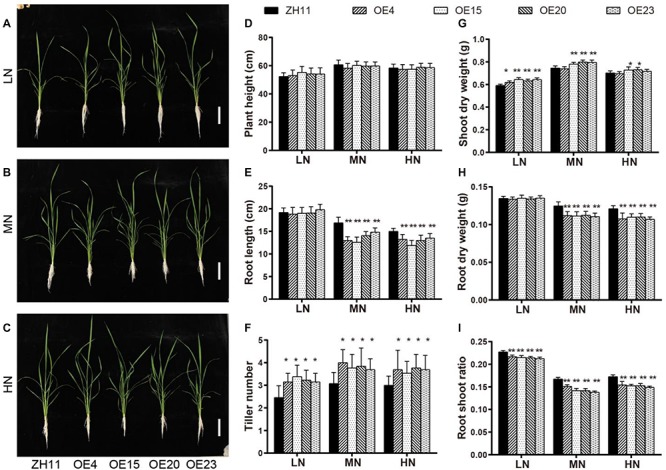
Performances of *OsMYB305*-overexpressing lines under different level of N supply. **(A–C)** Phenotype of *OsMYB305*-OE lines (OE4, OE15, OE20, OE23) and wild-type (ZH11) seedlings growing in hydroponic culture for 2 weeks. LN: 0.288 mM NH_4_NO_3_; MN:0.72 mM NH_4_NO_3_; HN:1.44 mM NH_4_NO_3_. **(D–I)** Statistic analysis of the growth parameters. Scale bars represents 10 cm. Values were means ± SD of 6 biological replications. Statistical significance was determined by Student’s *t*-test (*P < 0.05, **P < 0.01).

To assess the effect of *OsMYB305* overexpression on rice low N adaptation, we generated *OsMYB305-*overexpressing lines using a CaMV 35S promoter to drive its coding sequences. Four transgenic lines harboring > 200× more *OsMYB305* transcripts than the wild type (WT) (Supplementary Figures S1C,D) were selected to breed. Each generation was screened with hygromycin. The T2 seedlings of the *OsMYB305*-OE lines and the WT were hydroponically cultured under LN, MN and HN treatments (0.288, 0.72, and 1.44 mM NH_4_NO_3_, respectively) for 4 weeks (Figures 3A–C). Growth parameters were then measured and analyzed. Relative to the WT, the *OsMYB305*-OE lines had greater tiller numbers (28–37.4%, 19–33.3%, and 18–25.7%, respectively) at all three N levels (Figure 3F), and higher shoot dry weights (11.7–17.4% and 9.9–11.5%) under the LN and MN conditions (Figure 3G). In contrast, root length (12–25.4% and 10–20.8%) and root dry weight (10–11.4% and 9.1–11.5%) were significantly lower in the *OsMYB305*-OE lines under MN and HN conditions, respectively (Figures 3E,H). The root-shoot ratios were also decreased (4.7–6.5%, 9.7–17.5%, and 10.6–13.7%) for all three N treatments (Figure 3I). To ensure the reliability of these results, we repeated the hydroponic culture experiment under the same N regimes, and got very similar results (Supplementary Figures S3A–I). We also observed that the leaves of *OsMYB305*-OE lines drooped more severely compare to WT, and that the organs of flowers were developing normally while the pollen activities were slightly decreased via iodine dyeing (Supplementary Figure S4).

**FIGURE 4:**
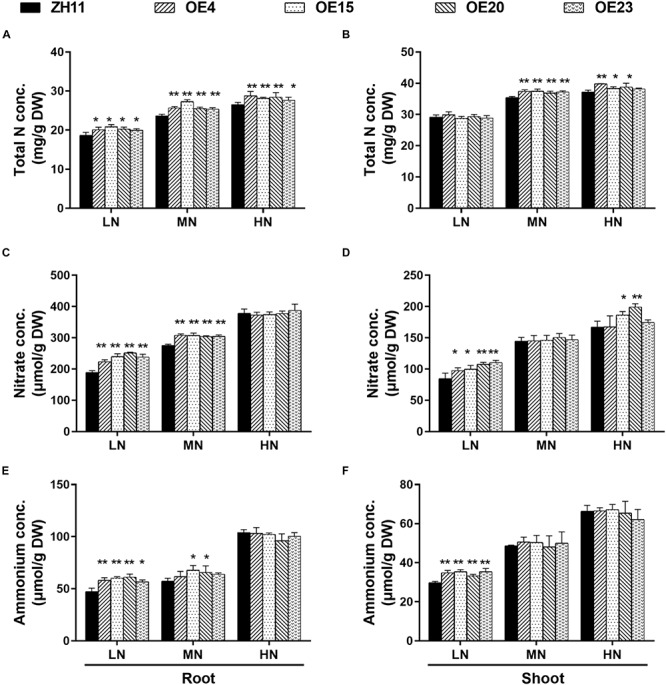
The concentrations of total N, nitrate and ammonium in *OsMYB305*-OE lines (OE4, OE15, OE20, OE23) and WT (ZH11). **(A,B)** Total N concentrations in the roots and shoots. **(C,D)** Nitrate concentrations in the roots and shoots. **(E,F)** Ammonium concentrations in the roots and shoots. Concs, concentrations; DW, dry weight. Values were means ± SD of 4 biological replications. The statistical significance was determined using the Student’s *t*-test (*P < 0.05, **P < 0.01).

To test whether *OsMYB305* overexpression influenced internal N status, the total N concentrations were measured. The total N concentrations in the roots of the *OsMYB305-*OE lines were 7.5–11.8%, 7.6–15.7%, and 4.1–8.7% higher than that of the WT under the LN, MN and HN conditions, respectively (Figure 4A). The shoot N concentrations were slightly higher than that of the WT (4.2–5.9% and 3–7%) under the MN and HN conditions (Figure 4B). The N concentration measurements were repeated, and the results from twice experiments were consistent (Supplementary Figures S3J,K). We further measured the concentrations of soluble proteins, nitrate, ammonium and free amino acids in roots and shoots of the rice seedling. The soluble protein levels did not differ between the *OsMYB305-*OE lines and the WT (Supplementary Figures S5A,B). However, the total free amino acids (Supplementary Figures S5C,D), nitrate (Figures 4C,D) and ammonium (Figures 4E,F) were increased in the roots and shoots of the *OsMYB305-*OE lines under the LN condition. Gln and Glu, which are direct products of N assimilation, were also significantly increased in the roots of the *OsMYB305-*OE lines under the LN condition (Supplementary Figures S5E–H). The total N concentrations in *OsMYB305-*OE lines and WT growing in the field (applying urea 250 kg/hm^2^) were measured, and the results showed that the total N concentrations in the straws of *OsMYB305-*OE lines were significantly higher than that of the WT, while the total N concentrations in the grains were undifferentiated (Supplementary Figure S14).

To confirm whether *OsMYB305* knockout resulted in opposite phenotype, we generated *OsMYB305* mutant lines via CRISPR/Cas9 technology. Six homozygotes were obtained with 1 bp insertions or deletions at the designated target site (Supplementary Figures S6A,B). The T2 seedlings were used to assess plant performance in hydroponic culture under the same nitrogen regime to which the *OsMYB305*-OE lines were subjected. However, the mutants were not phenotypically distinct from the WT. Moreover, there were no differences in total N concentration between the mutant lines and the WT (Supplementary Figure S6C).

### *OsMYB305* Overexpression Increases Nitrate Uptake Under Low N Condition

**FIGURE 5:**
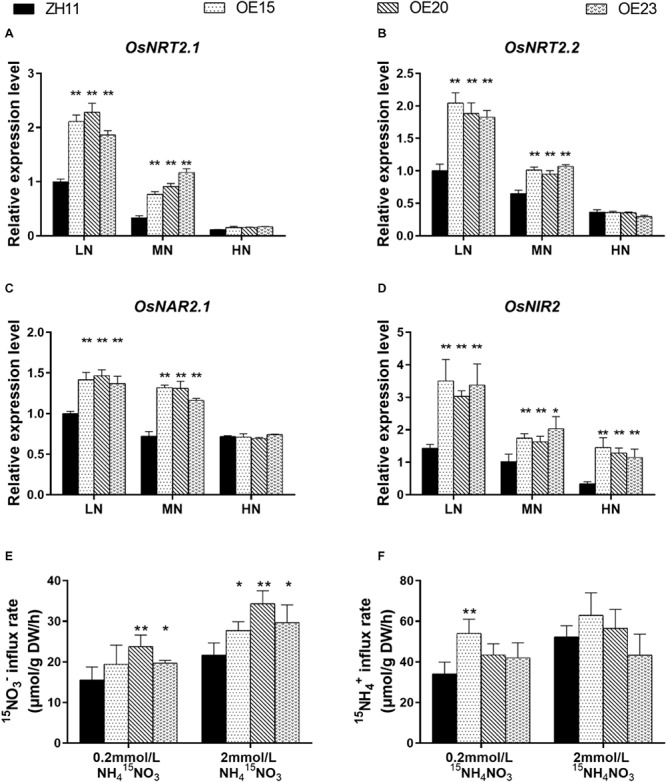
The expression level of N related genes and ^15^N uptake analysis. **(A–D)** The expression of *OsNRT2.1*, *OsNRT2.1*, *OsNAR2.1*, and *OsNIR2* in the roots of *OsMYB305*-OE lines (OE15, OE20, OE23) treating with various N level. **(E,F)** ^15^N influx rates of the *OsMYB305*-OE lines and WT (ZH11). The seedlings were grown in 0.2 or 2 mM NH_4_NO_3_ for 2 weeks, and then transferred to the medium in which the N sources were replaced by equal concentration of ^15^N labeled N sources for 10 min. NH_4_^15^NO_3_: NO_3_^–^ were labeled by ^15^N; ^15^NH_4_NO_3_: NH_4_^+^ were labeled by ^15^N. Data in **(A–D)** were means ± SD of 3 biological replications. Data in **(E,F)** were means ± SD of 4 biological replications. Statistical significance was determined by Student’s *t*-test (*P < 0.05, **P < 0.01).

As the 4 transgenic lines (OE4, OE15, OE20, OE23) showed consistent tendency in phenotype, total N and N compounds concentrations (Figures 3, 4), we used 3 lines (OE15, OE20, OE23) in the subsequent analyses. To explain why *OsMYB305* overexpression increases tissue N concentrations, we measured the expression levels of key genes involved in nitrogen uptake and assimilation. Three associated with the high-affinity nitrate transport system (*OsNRT2.1*, *OsNRT2.2*, and *OsNAR2.1*) and one encoding nitrite reductase (*OsNIR2*) was significantly up-regulated in the roots of *OsMYB305-*OE lines under LN and MN conditions (Figures 5A–D). In contrast, there were no significant differences between the OE lines and the WT in terms of the genes regulating ammonium uptake and assimilation (Supplementary Figure S7).

To test whether nitrate uptake was elevated in the *OsMYB305*-OE lines, a ^15^N trace analysis was conducted. Under 0.2 mM NH_4_^15^NO_3_, nitrate uptake was significantly increased by 26.6–52.7% in OE20 and OE23 (Figure 5E). Under 2 mM NH_4_^15^NO_3_, the nitrate influx rates significantly increased by 27.7–57.9% in OE15, OE20, and OE23 (Figure 5E). In nearly all treatments, however, the ammonium uptake rates did not differ between the *OsMYB305*-OE lines and the WT. The exception was OE15 for which the ammonium uptake had increased relative to the WT under 0.2 mM ^15^NH_4_NO_3_ (Figure 5F).

### *OsMYB305* Overexpression Inhibited Cellulose Synthesis in the Root

To elucidate the mechanism underlying the improved nitrogen uptake in the *OsMYB305*-OE lines, we used RNA-seq to analyze the gene expression profiles in the roots of OE15 and OE20 under low N condition. The data were deposited into the GEO database (GSE145579). Compared with the WT, 238 genes were down-regulated, and 227 genes were up-regulated (fold change > 1.5) in the roots of OE15 and OE20 (Supplementary Figures S8A,B). The differentially expressed genes (DEGs) were list in Supplementary Table S2. GO (gene ontology) analysis showed that the term “regulation of metabolic process” (GO:0019222) was significantly enriched in “biological process” among the up-regulated genes (Supplementary Figures S9A–C). Among the down-regulated genes, the GO terms “oxidation reduction” (GO:0055114), “carbohydrate metabolic process” (GO:0005975) and “response to stimulus” (GO:0050896) were significantly enriched in “biological process” and the GO term “catalytic activity” (GO:0003824) was enriched in “molecular function” (Supplementary Figures S8C,D).

**FIGURE 6:**
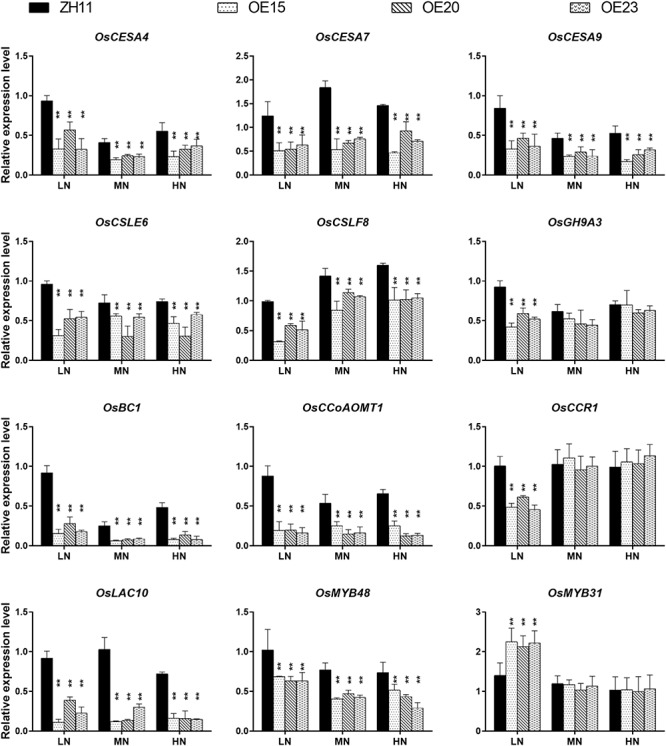
The expression level of cellulose and lignin synthesis related genes in the roots of *OsMYB305*-OE lines (OE15, OE20, OE23) and WT (ZH11) growing under various N treatments. Values were means ± SD of 3 biological replications. Statistical significance was determined using Student’s *t*-test (**P < 0.01).

A set of genes participating in secondary cell wall synthesis was down-regulated in the expression profiles (Table 1). Real-time q-PCR validated the expression levels of these genes. The expression of *OsCESA4*, *OsCESA7*, *OsCESA9*, *OsCSLF8*, *OsCSLE6*, *OsBC1*, and *OsGH9A3* (cellulose biosynthesis) and *OsMYB48* (positive regulator of secondary cell wall biosynthesis) were markedly down-regulated in the roots of *OsMYB305*-OE (Figure 6). In contrast, the expression of *OsMYB31* (negative regulator of secondary cell wall biosynthesis) was up-regulated under the LN condition (Figure 6). *OsCCR1*, *OsCCoAOMT* and *OsLAC10* (lignin biosynthesis) were also down-regulated (Figure 6). However, OsMYB305 could not activate or inhibit the promoters (2 kb) of *OsNRT2.1*, *OsNRT2.2*, *OsNAR2.1*, *OsCESA4*, *OsCESA7*, and *OsCESA9* in dual-luciferase reporter assay (Supplementary Figure S10).

**TABLE 1:** Differentially expressed genes involved in secondary cell wall biosynthesis in *OsMYB305*-OE lines (OE15, OE20).

**Gene name**	**MSU accession number**	**OE15 log2(fold change)**	**OE20 log2(fold change)**	**Functional annotation**
*OsCESA4*	LOC_Os01g54620	−0.81	−0.56	Cellulose synthase
*OsCESA7*	LOC_Os10g32980	−0.79	−0.56	Cellulose synthase
*OsCESA9*	LOC_Os09g25490	−0.93	−0.76	Cellulose synthase
*OsCSLF8*	LOC_Os07g36630	−0.93	−0.78	Cellulose synthase-like family F
*OsCSLE6*	LOC_Os09g30130	−0.98	−0.6	Cellulose synthase-like family E
*OsBC1*	LOC_Os03g30250	−0.84	−0.48	COBRA-like protein precursor
*OsGH9A3*	LOC_Os03g52630	−0.55	−0.47	Endoglucanase, putative
*OsLAC10*	LOC_Os03g16610	−0.88	−1.02	Laccase precursor protein
*OsLAC14*	LOC_Os07g01110	−0.75	−0.56	Laccase precursor protein
*OsCCR1*	LOC_Os01g18110	−0.75	−0.6	Cinnamoyl CoA reductase
*OsCCoAOMT*	LOC_Os08g38910	−0.46	−0.61	Caffeoyl-CoA O-methyltransferase
*OsMYB31*	LOC_Os09g36730	0.61	0.49	MYB family transcription factor

**FIGURE 7:**
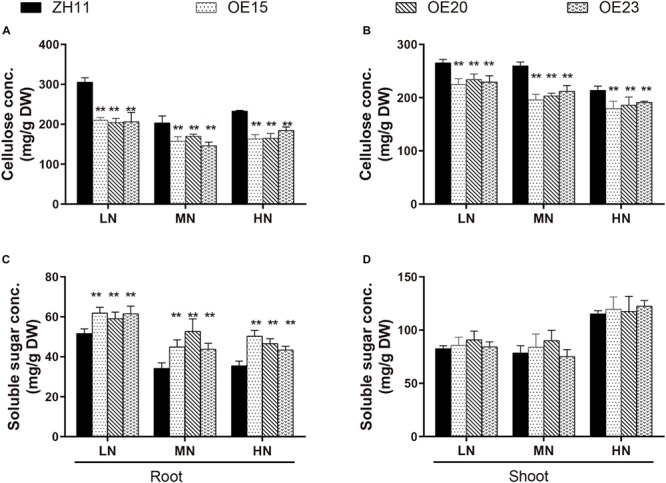
The concentrations of cellulose and soluble sugar in the *OsMYB305*-OE lines (OE15, OE20, OE23) and WT (ZH11) growing under various N treatments. **(A,B)** The cellulose concentrations in the roots and shoots of the *OsMYB305*-OE lines and WT. **(C,D)** The soluble sugar concentrations in the roots and shoots of the *OsMYB305*-OE lines and WT. Values were means ± SD of 3 biological replications. Statistical significance was determined using Student’s *t*-test (**P < 0.01).

Cell wall compositions were then examined and compared. Relative to the WT, the cellulose concentrations in the roots of OE15, OE20 and OE23 were 31–33%, 16.5–28.2%, and 20.7–29.6% lower under the LN, MN, and HN conditions, respectively (Figure 7A). The cellulose concentrations in the shoots of OE15, OE20 and OE23 were also 11.8–15.2%, 18.5–24.6%, and 10.6–16% lower compared with the WT under the three N levels, respectively (Figure 7B). However, neither the hemicellulose nor the lignin content differed between the *OsMYB305*-OE lines and the WT (Supplementary Figures S11A–D). In addition, the lignification degrees in the roots, leaf sheaths and leaf blades of OE15 and OE20 were obviously weaker than that in WT by phloroglucinol dying using freehand sections (Supplementary Figure S12).

### *OsMYB305* Overexpression Increased Soluble Sugar Concentration and Altered Carbohydrate Metabolism

Cellulose synthesis requires UDP-glucose as a substrate. Thus, we measured the carbohydrates concentrations in the *OsMYB305*-OE lines. The total C concentrations did not differ between the OE lines and the WT under the LN and HN conditions (Supplementary Figures S13A,B). Therefore, we measured the soluble sugar and starch concentrations. The starch concentration was slightly elevated in the roots of the *OsMYB305*-OE lines under LN condition (Supplementary Figures S13C,D). The soluble sugar concentrations were significantly increased by 14.3–19.9%, 21.9–54.1%, and 22.5–41.9% in the roots of the *OsMYB305*-OE lines under the LN, MN, and HN conditions, respectively (Figure 7C). However, the soluble sugar concentrations in the shoots did not significantly differ between the *OsMYB305*-OE lines and the WT (Figure 7D). Moreover, relative to the WT, the sucrose and glucose concentrations were significantly increased in the roots of the *OsMYB305*-OE lines under the LN, MN, and HN conditions but the fructose concentration did not change (Supplementary Figures S13E–G).

We further used LC-MS/MS to determine whether carbon metabolism changed in the roots of *OsMYB305*-OE lines under LN condition. Relative to the WT, the glycolysis metabolites G-1-P, G-6-P, F-6-P, and PEP were significantly decreased in the roots of the OE lines (Table 2). In contrast, the tricarboxylic acid (TCA) cycle intermediates before 2-oxoglutarate (2OG) including citrate, aconitate and isocitrate were increased in the roots of the OE lines. However, the TCA cycle intermediates after 2OG including fumarate and malate were decreased in the roots of the OE lines (Table 2). In addition, the ATP concentration and the NADPH/NADP^+^ ratio were increased in the roots of the OE lines under the LN condition (Table 2).

**TABLE 2:** Metabolites concentrations in the roots of *OsMYB305*-OE lines (OE15, OE20, OE23) and WT (ZH11) under LN condition.

	**ZH11**	**OE15**	**OE20**	**OE23**
Glyoxylate	6.22 ± 0.37	4.79 ± 0.33**	4.32 ± 0.1**	4.86 ± 0.43**
G-6-P	370.49 ± 42.4	285.22 ± 16**	292.13 ± 3.97**	238.34 ± 7.67**
F-6-P	389.7 ± 44.25	300.17 ± 15.26**	306.8 ± 3.91**	250.35 ± 7.91**
G-1-P	401.86 ± 45.03	310.14 ± 16.23**	318.58 ± 4**	257.58 ± 8.42**
PEP	0.58 ± 0.16	0.3 ± 0.09**	0.22 ± 0.09**	0.14 ± 0.02**
ADPG	1.54 ± 0.44	0.66 ± 0.13**	0.83 ± 0.17**	0.77 ± 0.14**
Citrate	542.76 ± 12.33	763.91 ± 84.57**	638.57 ± 28.31**	719.54 ± 74.18**
Isocitrate	639.13 ± 15.38	815.61 ± 80.3**	723.38 ± 39.84**	823.79 ± 47.32**
Aconitate	2.47 ± 0.24	4.09 ± 0.39**	3.7 ± 0.4**	4.21 ± 0.16**
Malate	620.86 ± 36.76	430.89 ± 22.68**	429.67 ± 10.52**	473.51 ± 40.22**
Fumarate	163.31 ± 9.39	123.04 ± 7.4**	112 ± 2.87**	122.32 ± 9.54**
ATP	4.59 ± 0.86	6.64 ± 0.3**	8.62 ± 1.33**	6.41 ± 0.43**
F2,6BP	5.9 ± 1.11	6.12 ± 0.1	7.13 ± 1.36*	7.4 ± 0.37*
NADPH/NADP +	54.7 ± 17.16	159.65 ± 14.34	275.63 ± 31.63**	306.5 ± 35.64**

Values were means ± SD of 3 biological replications. The unit of measurement is nmol g^–1^ (FW). Statistical significance was determined by Student’s t-test (*P < 0.05; **P < 0.01). ND: not detect.

**FIGURE 8:**
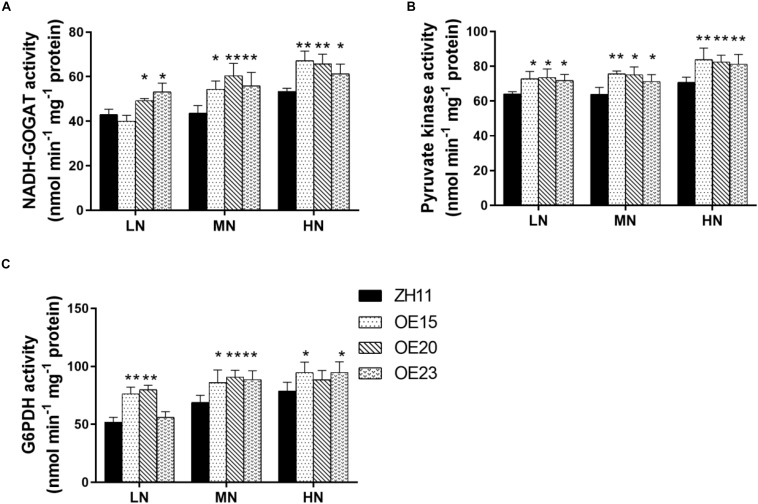
The enzyme activities in the roots of *OsMYB305*-OE (OE15, OE20, OE23) and WT (ZH11) under different N treatments. Values were means ± SD of 3 biological replications. Statistical significance was determined by Student’s *t*-test (*P < 0.05, **P < 0.01).

**FIGURE 9:**
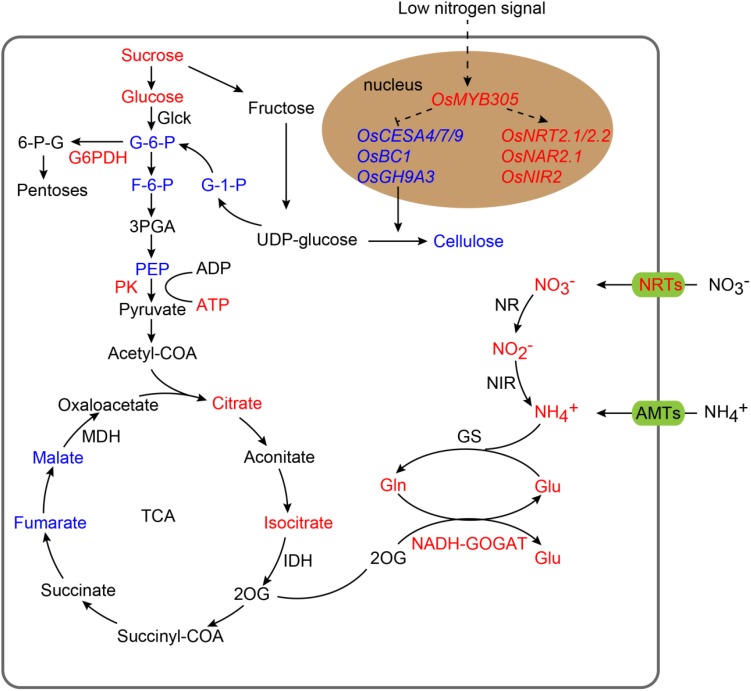
The integrated schematic diagram of the changes on gene expression, metabolism and enzyme activities in *OsMYB305*-OE lines. Increased objects were represented by red letters; decreased objects were represented by blue letters.

We further detected the activity of key enzymes in carbon metabolism and N assimilation including NR, NIR, GS, NADH-GOGAT, Glc-K, Fru-K, PEPC, Pyr-K, ICDH, and G6PDH. The activity levels of NADH-GOGAT, Pyr-K, and G6PDH were significantly increased in the roots of the *OsMYB305*-OE lines under the LN, MN and HN conditions (Figures 8A–C). To exhibit the effects of *OsMYB305* overexpression more systematically, we integrated the alterations on gene expression, metabolism and enzymatic activities into a schematic diagram (Figure 9).

## Discussion

Transcription factors have been considered potential targets for the improvement of crop NUE. Several studies have identified numerous transcription factors in rice that respond to low-nitrogen stress. However, their functions were poorly understood. In this work, we identified a novel transcription factor gene *OsMYB305*. Its expression was induced by low-nitrogen conditions. We evaluated its potential roles in nitrogen uptake and assimilation using its overexpression lines.

### *OsMYB305* Overexpression Improved Rice Growth Under Low Nitrogen Condition by Facilitating Nitrate Uptake

OsMYB305 belongs to the R2R3-MYB transcription factor family. It has high homology with AmMYB305, ZmMYB305, NtMYB305 and the AtMYB21-clade proteins AtMYB21, AtMYB24, and AtMYB57 (Figure 2A). In *Antirrhinum majus*, AmMYB305 regulates the phenylpropanoid biosynthetic pathway (Sablowski et al., 1994). In *Arabidopsis*, AtMYB21, AtMYB24, and AtMYB57 regulate stamen and pollen maturation via the jasmonate pathway (Cheng et al., 2009; Mandaokar and Browse, 2009; Song et al., 2011). NtMYB305 plays an important role in primary carbohydrate metabolism and affects nectar production in tobacco (*Nicotiana tabacum* L. cv. TN90) (Liu et al., 2009; Liu and Thornburg, 2012; Wang et al., 2014). Though *OsMYB305* was preferentially expressed in the young panicles (Supplementary Figures S1A,B), its transcript was induced by N-deficiency in the roots (Figures 1A–D). In the unicellular red alga *Cyanidioschyzon merolae*, *CmMYB1* expression was induced by nitrogen depletion. The product it encoded bound the promoters of several key nitrogen assimilation genes including *CmNRT*, *CmNIR*, and *CmGS.* In this way, inorganic nitrogen uptake was stimulated (Imamura et al., 2009). *OsENOD93-1* significantly responded to N induction and reduction. Plants overexpressing this gene accumulated comparatively higher total amino acid and N concentrations in their roots and presented with relatively greater shoot dry biomass and seed yield (Bi et al., 2009). Thus, the response of *OsMYB305* to N-deficiency implied that it may has a potential role in low N adaptation.

*OsMYB305*-overexpressing plants showed relatively higher tiller numbers and shoot dry weights than that of the WT under low- and moderate N conditions (Figures 3F,G). In contrast, their root lengths and root dry weights were significantly decreased and their root-shoot ratios were lower under moderate- and high N conditions (Figures 3E,H,I). Low root to shoot ratio implies adequate internal N status (Beier et al., 2018). Further analysis indicated that the *OsMYB305*-OE lines had relatively higher N concentrations (Figures 4A,B) and levels of nitrogen-containing compounds such as nitrate, ammonium (Figures 4C–F), Gln and Glu (Supplementary Figures S5E–H).

Gene expression analysis revealed that the transcription of *OsNRT2.1*, *OsNRT2.2*, *OsNAR2.1*, and *OsNIR2* was significantly up-regulated in the roots of *OsMYB305-*OE lines under the LN and MN conditions (Figure 5A). OsNRT2.1, OsNRT2.2, and OsNRT2.3a interact with OsNAR2.1 in a high-affinity nitrate transport system in rice (Yan et al., 2011; Liu et al., 2014). Up-regulated expression of *OsNRT2.1* driven by the *OsNAR2.1* promoter significantly improved rice yield and NUE (Chen et al., 2016). The overexpression of *OsNAR2.1* driven by the native *OsNAR2.1* promoter also increased NO_3_^–^ and NH_4_^+^ uptake in rice roots (Chen et al., 2017). ^15^N trace analysis disclosed that nitrate influx in the *OsMYB305-*OE lines was significantly increased under low- and high-nitrogen conditions (Figure 5E). Only OE15 presented a significant increase in ^15^NH_4_^+^ uptake under 0.2 mM ^15^NH_4_NO_3_ (Figure 5F). Nevertheless, the ammonium concentrations in the roots and shoots of the *OsMYB305-*OE lines were significantly higher than those for the WT under low-N condition (Figures 4E,F). These results suggest that ammonium uptake was elevated in the *OsMYB305-*OE rice lines. The ^13^N radiotracer technique showed that the net ammonium uptake was comparatively higher when nitrate was present along with the ammonium (Kronzucker et al., 1999). This phenomenon was reported and corroborated in several subsequent studies (Duan et al., 2006; Li et al., 2006; Fan et al., 2016; Chen et al., 2017) and may partially account for the observed results for the *OsMYB305-OE* lines. Overall, our results indicated that *OsMYB305* overexpression improved rice growth under low nitrogen conditions by enhancing nitrogen uptake.

The phenotypes of the *OsMYB305* mutant lines were not distinct from that of the WT at the various N levels. Furthermore, their internal N concentrations did not differ from each other. One possible explanation for this phenomenon is the functional redundancy of the homologous genes (Supplementary Figure S2). However, this hypothesis must be empirically tested and validated.

### OsMYB305 May Coordinate N Assimilation and Secondary Cell Wall Biosynthesis

Transcriptome data showed that *OsMYB305* overexpression repressed the genes associated with lignocellulose biosynthesis (Table 1). *OsCESA4*, *OsCESA7*, *OsCESA9*, *OsBC1*, and *OsGH9A3* play central roles in cellulose biosynthesis (Li et al., 2003; Tanaka et al., 2003; Zhang et al., 2009; Kotake et al., 2011; Xie et al., 2013; Wang et al., 2016; Li F. C. et al., 2017). These genes are conservatively controlled by the MYB transcription factors. OsMYB48/20, OsMYB58/63, OsMYB55/61 and OsMYB103/103L act as positive regulators (Huang et al., 2015; Noda et al., 2015; Ye et al., 2015; Rao and Dixon, 2018; Ye et al., 2018). On the other hand, the AtMYB4-clade protein homolog OsMYB31 functions as a negative regulator (Fornalé et al., 2010; Agarwal et al., 2016). Here, the expression of *OsCESA4*, *OsCESA7*, *OsCESA9*, *OsBC1*, *OsGH9A3*, and *OsMYB48* was down-regulated in the roots of the *OsMYB305-*OE lines. In contrast, the expression of *OsMYB31* was up-regulated (Figure 6). *OsCCR1*, *OsCCoAOMT*, and *OsLAC10* encoding enzymes involved in lignin biosynthesis were also down-regulated (Figure 6). A cell wall component analysis revealed that in comparison to the WT, the cellulose concentrations were significantly reduced in the *OsMYB305*-OE lines (Figures 7A,B). However, the hemicellulose and lignin concentrations in the *OsMYB305*-OE lines did not differ from those of the WT (Supplementary Figure S11). Overall, *OsMYB305* overexpression inhibited cellulose biosynthesis.

Secondary cell wall (structural carbohydrate) formation is associated with numerous biological processes and determined by the sugar levels in plants (Rao and Dixon, 2018). High nitrogen fertilizer levels down-regulated the expression of lignin biosynthesis genes in rice and decreased their shoot cellulose and lignin content (Zhang et al., 2017). OsGLU3 is a putative membrane-bound endo-1,4-β-glucanase. It regulates nitrogen and phosphate starvation-induced root elongation. Its loss-of-function mutant presented with shorter roots and lower cellulose content under low nitrogen or phosphate conditions compared with the WT (Zhang et al., 2012a, b). *OsUGE-1* encodes a UDP-glucose-4-epimerase and responds to exogenous N supply. Its overexpressing plants display relatively high sucrose concentrations and low cellulose content under N-limited condition (Guevara et al., 2014). Thus, there is a close relationship between N assimilation and secondary cell wall biosynthesis. However, the molecular mechanisms remain unclear. Here, we showed that *OsMYB305* overexpression enhanced N uptake and inhibited cellulose biosynthesis. Our findings confirmed the association between N assimilation and secondary cell wall formation and OsMYB305 may coordinate these processes.

### *OsMYB305* Overexpression Altered Carbohydrate Metabolism

In plants, N assimilation and C metabolism are closely linked biological processes. Inorganic nitrogen assimilation requires carbon skeletons derived from glycolysis and the TCA cycle. Thus, carbon metabolism must be regulated to meet carbon and energy needs under various N conditions. On the other hand, photosynthetic products affect the expression of genes regulating nitrogen uptake and assimilation (Sun et al., 2013; Rubio-Asensio and Bloom, 2017). Sucrose and glucose activate the nitrogen transport systems in *Arabidopsis* roots by up-regulating the expression of *NRT2* and *AMT1* genes (Lejay et al., 2003). Up-regulated expression of *AtNRT2.1* stimulated by glucose depends on hexokinase (HXK) activity and pentose phosphate pathway (PPP) metabolism (Lejay et al., 2008; Bussell et al., 2013; de Jong et al., 2014). Overexpression of the hexose transporter *STP13* increased the endogenous sucrose content, up-regulated *NRT2.2* expression, and enhanced nitrate uptake in *Arabidopsis* under low-N condition (Schofield et al., 2009). In rice, exogenous sucrose supplementation increased the accumulation of *OsNRT2.1*, *OsNRT2.2*, *OsNRT2.3*, and *OsNAR2.1* transcripts (Feng et al., 2011). These results suggest a conserved molecular mechanism underlying the increase in nitrate uptake promoted by C metabolites. In this study, *OsMYB305* overexpression increased the soluble sugar (sucrose and glucose) concentrations in rice roots (Figure 7C and Supplementary Figures S13E,F). The expression of *OsNRT2.1*, *OsNRT2.2*, *OsNAR2.1*, and *OsNIR2* was markedly up-regulated (Figures 4A–D) and nitrogen uptake was enhanced under low-N condition (Figures 4E,F).

Metabolites in glycolysis and the tricarboxylic acid cycle (TCA) were significantly altered in the roots of the *OsMYB305*-OE lines (Table 2). NADH-GOGAT, Pyr-K, and G6PDH participating in N assimilation, the TCA cycle, and the pentose phosphate pathway (OPPP) were increased in the roots of the *OsMYB305*-OE lines (Figure 8). In heterotrophic plastids, OPPP generates NADPH which reduces ferredoxin as the electron donor for nitrite reductase (NiR) (Noctor and Foyer, 1998; Masakapalli et al., 2013). In the roots of the *OsMYB305*-OE lines, the NADPH/NADP^+^ ratio increased by 3–6 × (Table 2). Glycolysis and TCA are basic processes in C metabolism and furnish C skeletons and chemical energy for ammonium assimilation. Enhanced ammonium assimilation usually results in comparatively higher amino acid and protein levels (Fang et al., 2013; Sato and Yanagisawa, 2014; Bao et al., 2015; Chen et al., 2017). Relative to the WT, the total N and free amino acid concentrations were significantly increased in the *OsMYB305*-OE lines under low-nitrogen condition (Supplementary Figures S5C–H). The present study corroborates previous reports by disclosing that increased C availability and enhanced C metabolism improve nitrogen uptake and assimilation in rice.

### *OsMYB305* Is an Attractive Candidate for Improving NUE in Rice

Here, we demonstrated that compared with the WT, transgenic plants overexpressing *OsMYB305* exhibited relatively greater tiller number, shoot dry weight, total N concentration and nitrogen influx rate under low-N condition. Thus, *OsMYB305* is a potential target for NUE improvement in rice. However, these results were derived from hydroponic culture in a greenhouse maintaining a constant and stable growth environment for the rice. In the future, the effects of *OsMYB305* overexpression on rice NUE should also be evaluated in field trials. We showed that *OsMYB305* encoded a transcription activator (Figures 2B–D) and *OsMYB305* overexpression extensively altered the gene expression levels in rice roots (Supplementary Figures S8A,B). In this study, we did not identify the genes that are direct targets of OsMYB305. For this reason, a vital future research objective is the elucidation of the OsMYB305 transcriptional regulatory network. Based on the present results, we concluded that the repression of cellulose synthesis increased non-structural carbohydrate (sucrose and glucose) production and content, enhanced carbon metabolism and, by extension, stimulated nitrogen uptake and assimilation. The present study proposes an innovative strategy for the improvement of nitrogen uptake in rice by reducing carbohydrate flux toward cell wall biosynthesis. Future research should also endeavor to determine whether this mechanism is common in various crops.

## Data Availability Statement

All datasets generated for this study are included in the article/Supplementary Material. The raw data of transcriptome can be found in GEO database (GSE145579).

## Author Contributions

XL and DW: experimental design. DW, TX, ZY, HG, and LL: experiments. DW: data analysis. XL, DW, and MY: manuscript preparation. XL and HC: supervision, funding, and reagents.

## Conflict of Interest

The authors declare that the research was conducted in the absence of any commercial or financial relationships that could be construed as a potential conflict of interest.

## Footnotes

1 https://www.ncbi.nlm.nih.gov/

2 http://crispr.hzau.edu.cn/CRISPR/
