# Supplementary material for: Overexpression of OsMYB305 in Rice Enhances the Nitrogen Uptake Under Low-Nitrogen Condition
Source: Front Plant Sci. 2020 Apr 15;11:369. doi: 10.3389/fpls.2020.00369 (PMC7174616; doi:10.3389/fpls.2020.00369)
Supplement: Supplementary file 1 [file Data_Sheet_1.PDF]

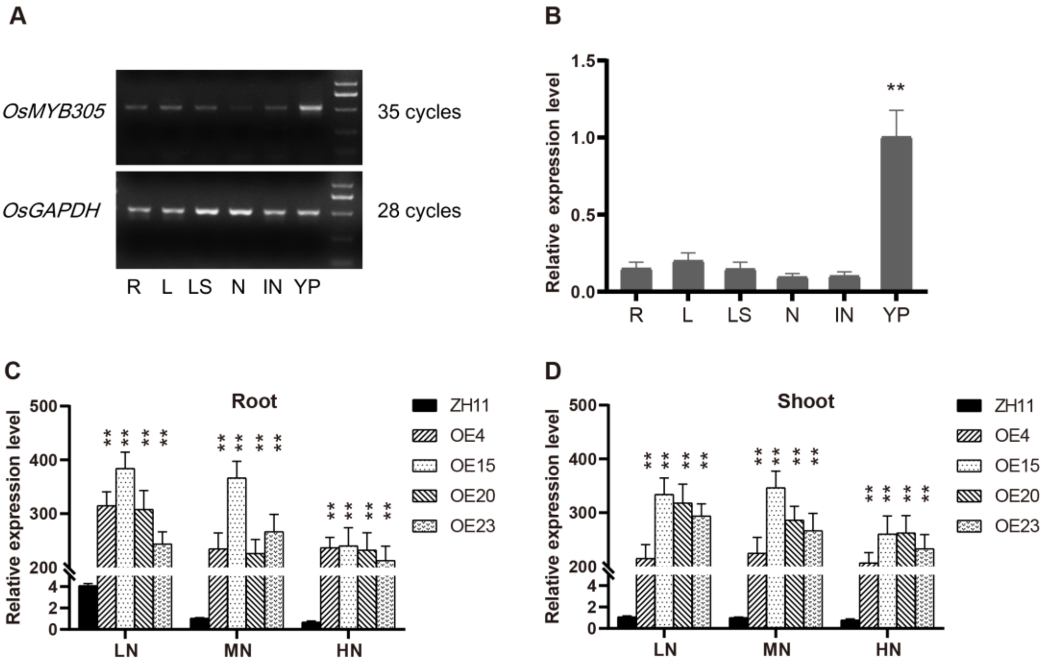

Figure S1. The expression level of *OsMYB305* in various tissues and transcriptional examination of *OsMYB305*-OE lines (OE4, OE15, OE20, OE23). **(A, B)** The expression of *OsMYB305* in various tissues were measured using RT-PCR and realtime q-PCR. **(C, D)** The expression level of *OsMYB305* in the roots and shoots of *OsMYB305*-OE lines and WT (ZH11). R: root; L: leaves; LS: leaf sheath; N: node; IN: internode; YP: young panicle. Data were means ± SD of 3 biological replications. Statistical significance was determined by Student's *t*-test (**$P < 0.01$).

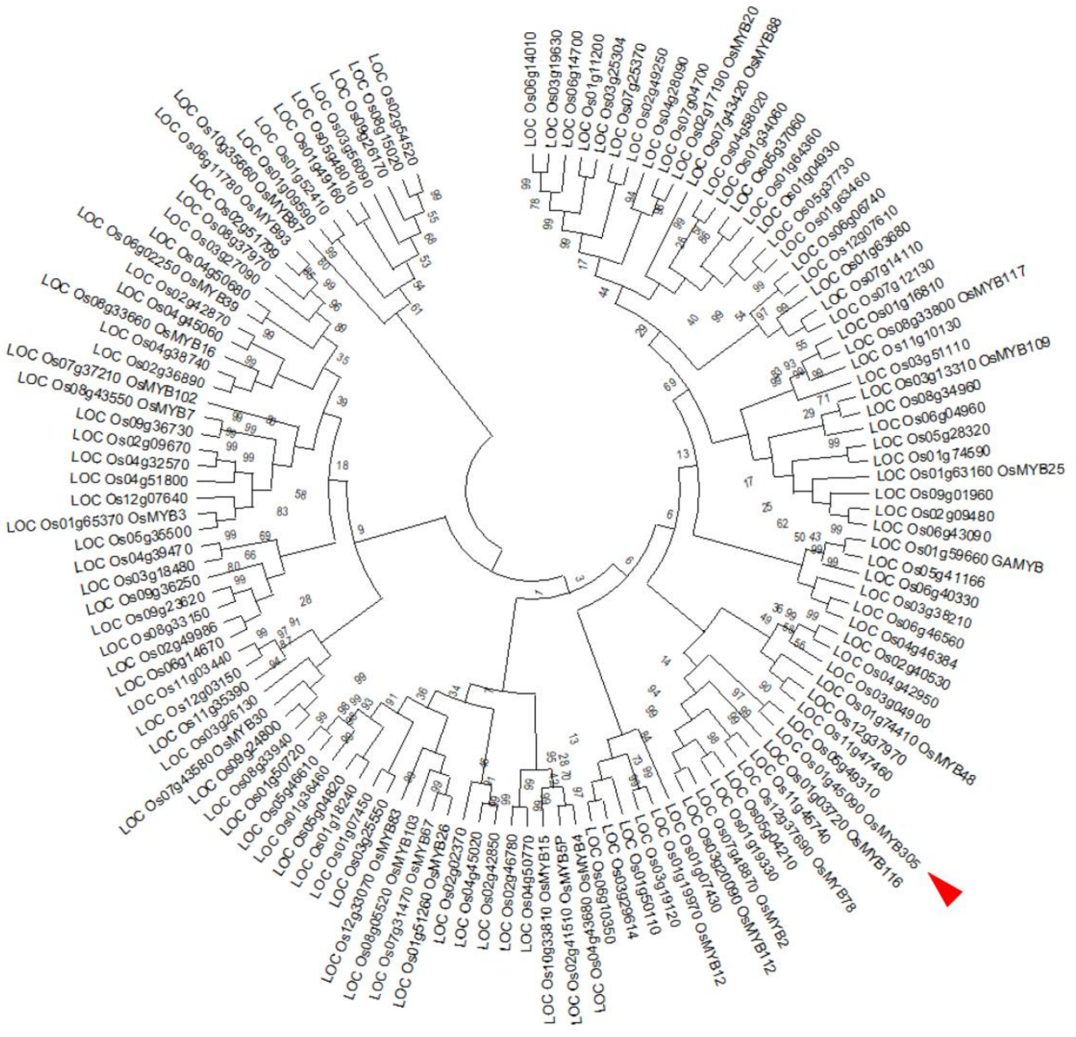

Figure S2. Phylogenetic analysis of the R2R3-MYB transcription factor family in rice. The protein sequences of 125 R2R3-MYB transcription factors were download from the article (Zhao et al., 2014). The red arrow directed OsMYB305.

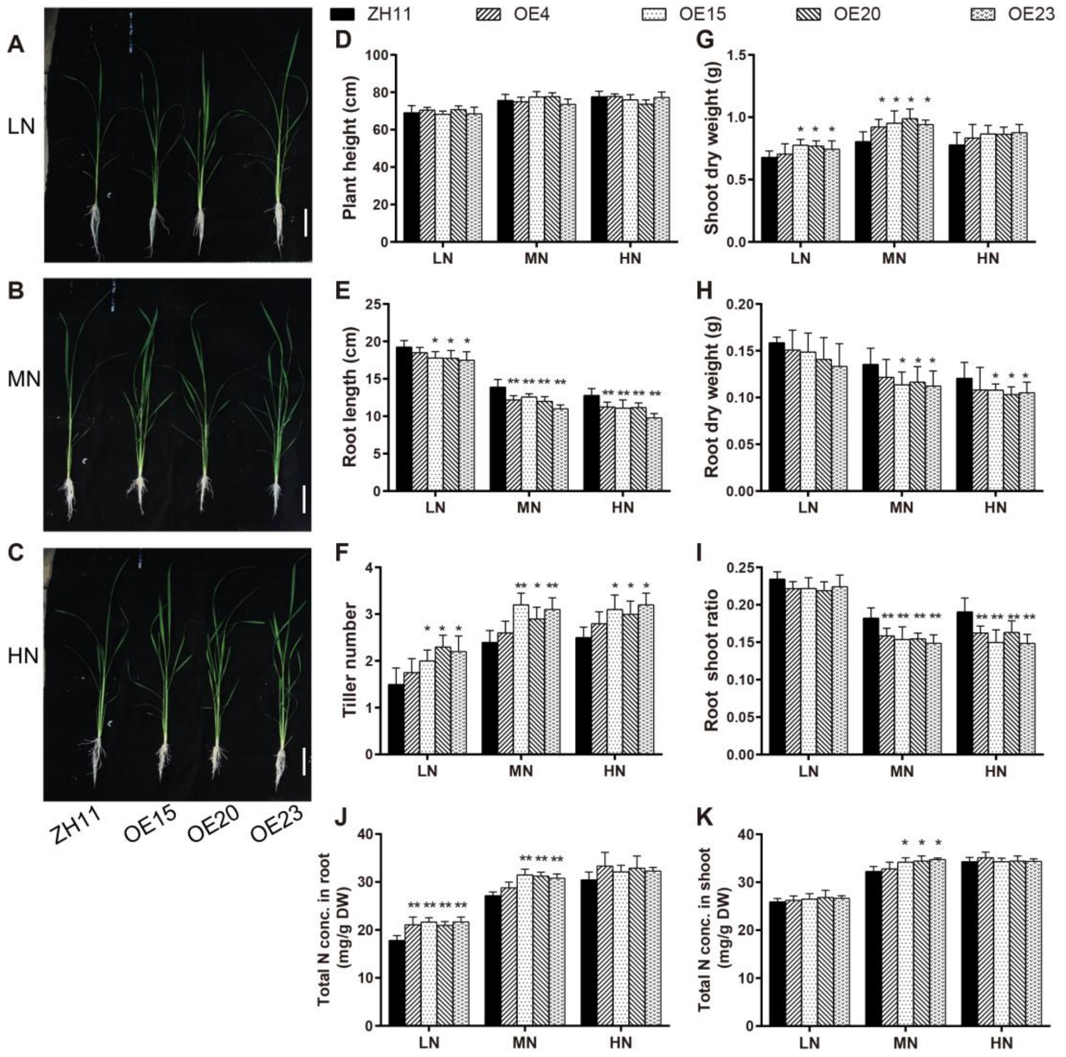

Figure S3. Phenotypic statistics and N concentrations measurement of the *OsMYB305*-OE lines (OE4, OE15, OE20, OE23) and WT (ZH11) under various N supplies. **(A-C)** The photographs of *OsMYB305*-OE lines and WT growing in hydroponic culture for 2 weeks. LN: 0.288 mM NH_4NO_3; MN:0.72 mM NH_4NO_3; HN:1.44 mM NH_4NO_3. **(D-I)** Statistics of the growth parameters. **(J, K)** Total N concentrations in roots and shoots of the *OsMYB305*-OE lines and WT. Scale bars represent 10 cm. Data were means ± SD of 6 biological replicates. Statistical significance was determined by Student's *t*-test (**P < 0.05*, ***P < 0.01*).

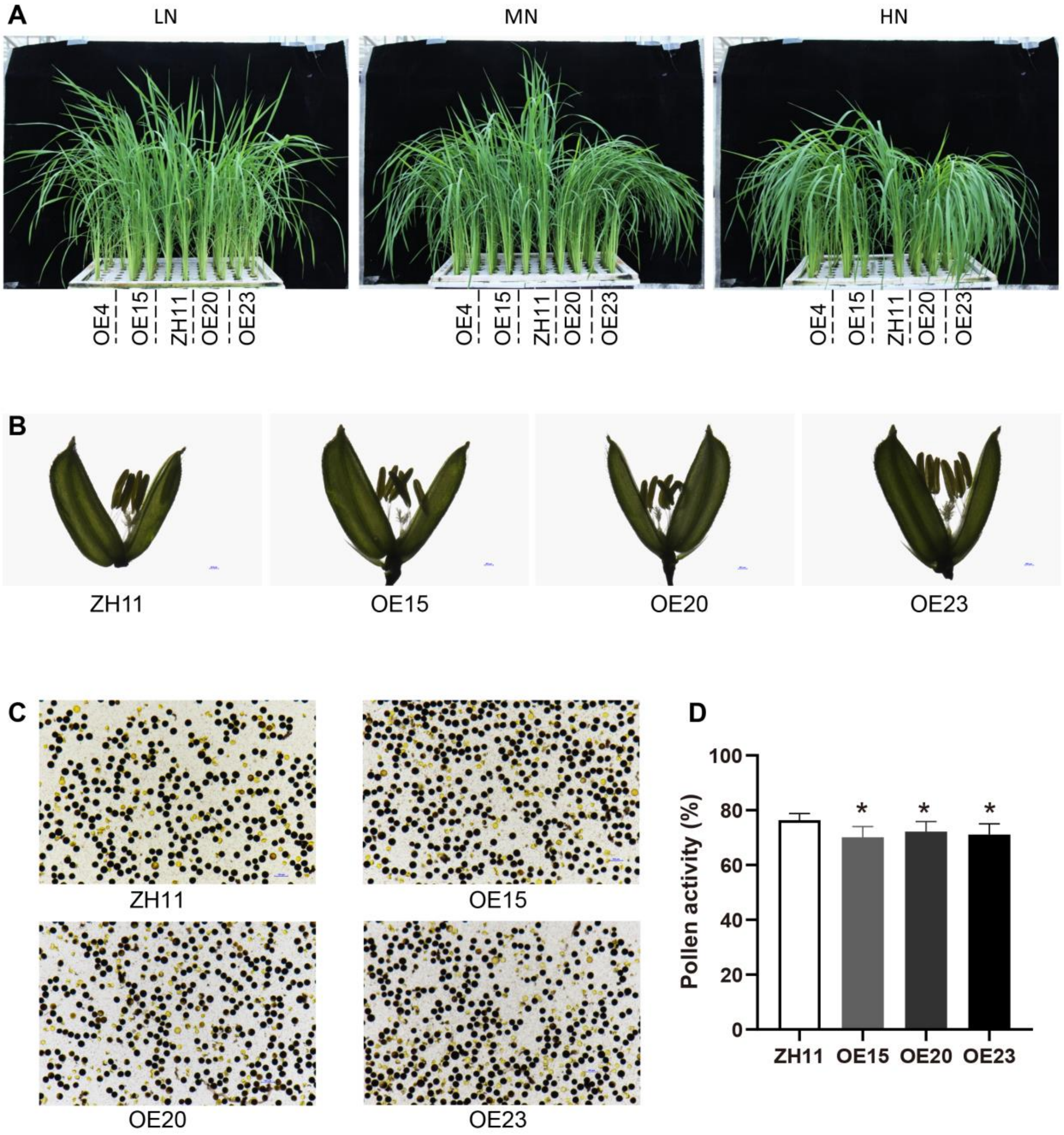

Figure S4. Phenotypes on leaves and spikelet of the *OsMYB305*-OE lines (OE4, OE15, OE20, OE23) and WT (ZH11). **(A)** The phenotypes on leaves of the *OsMYB305*-OE lines and WT under various N treatments. **(B)** Phenotypes on the spikelet of *OsMYB305*-OE lines and WT. **(C)** Photographs of the pollen grains dying by iodine. **(D)** The statistics of pollen activities. Data were means ± SD of 10 biological replicates. Statistical significance was determined by Student's *t*-test (*P < *0.05*).

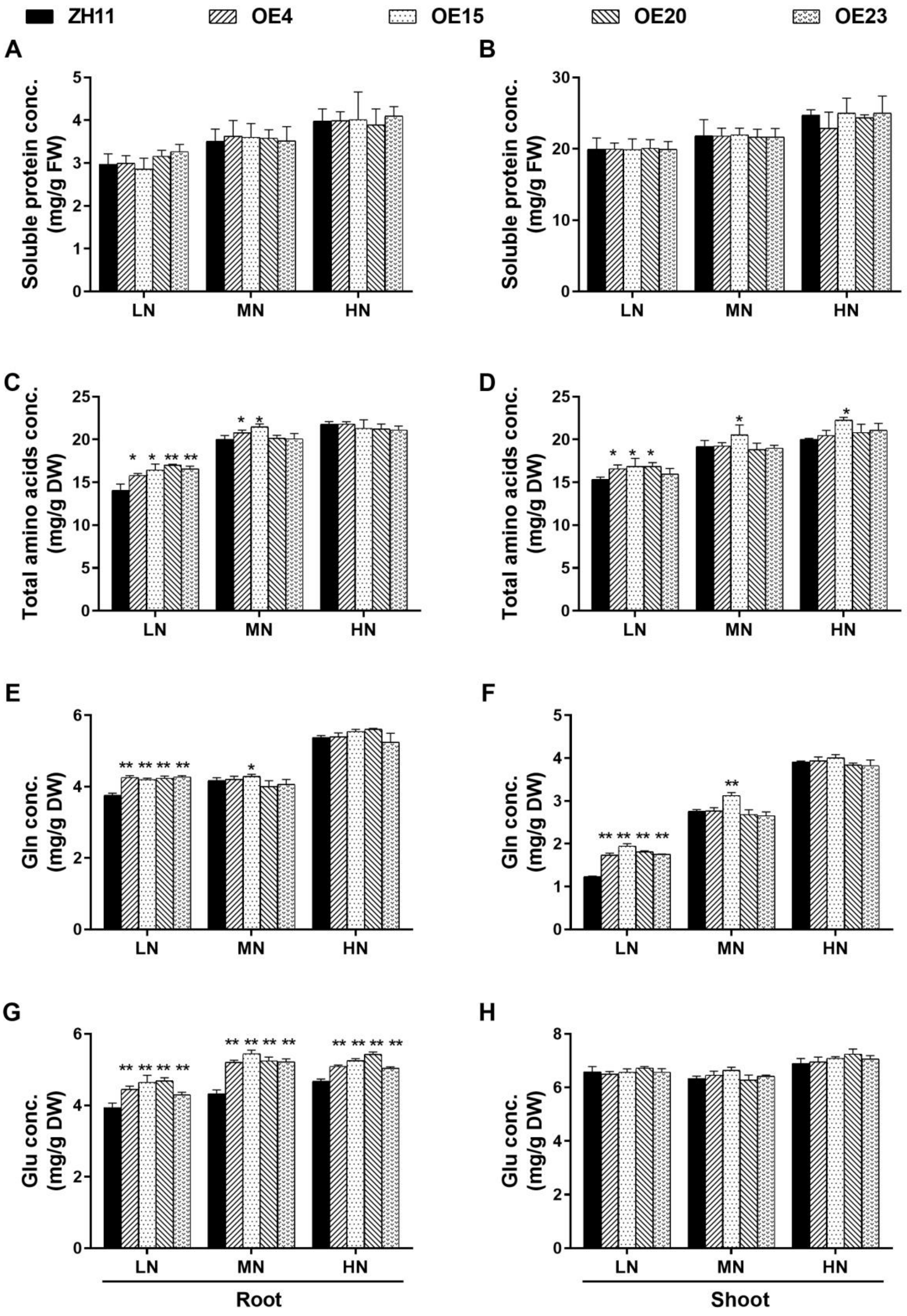

Figure S5. The concentrations of N compounds in *OsMYB305*-OE lines (OE4, OE15, OE20, OE23) and WT (ZH11). **(A，B)** Soluble protein concentrations in the roots and shoots. **(C, D)** Total free amino acids concentrations in the roots and shoots. **(E, F)** Free Gln concentrations in the roots and shoots. **(G, H)** Free Glu concentrations in the roots and shoots. Conc.: concentrations. Data were means ± SD of 3 biological replicates. Statistical significance was determined by Student's *t*-test (* $P < 0.05$, ** $P < 0.01$).

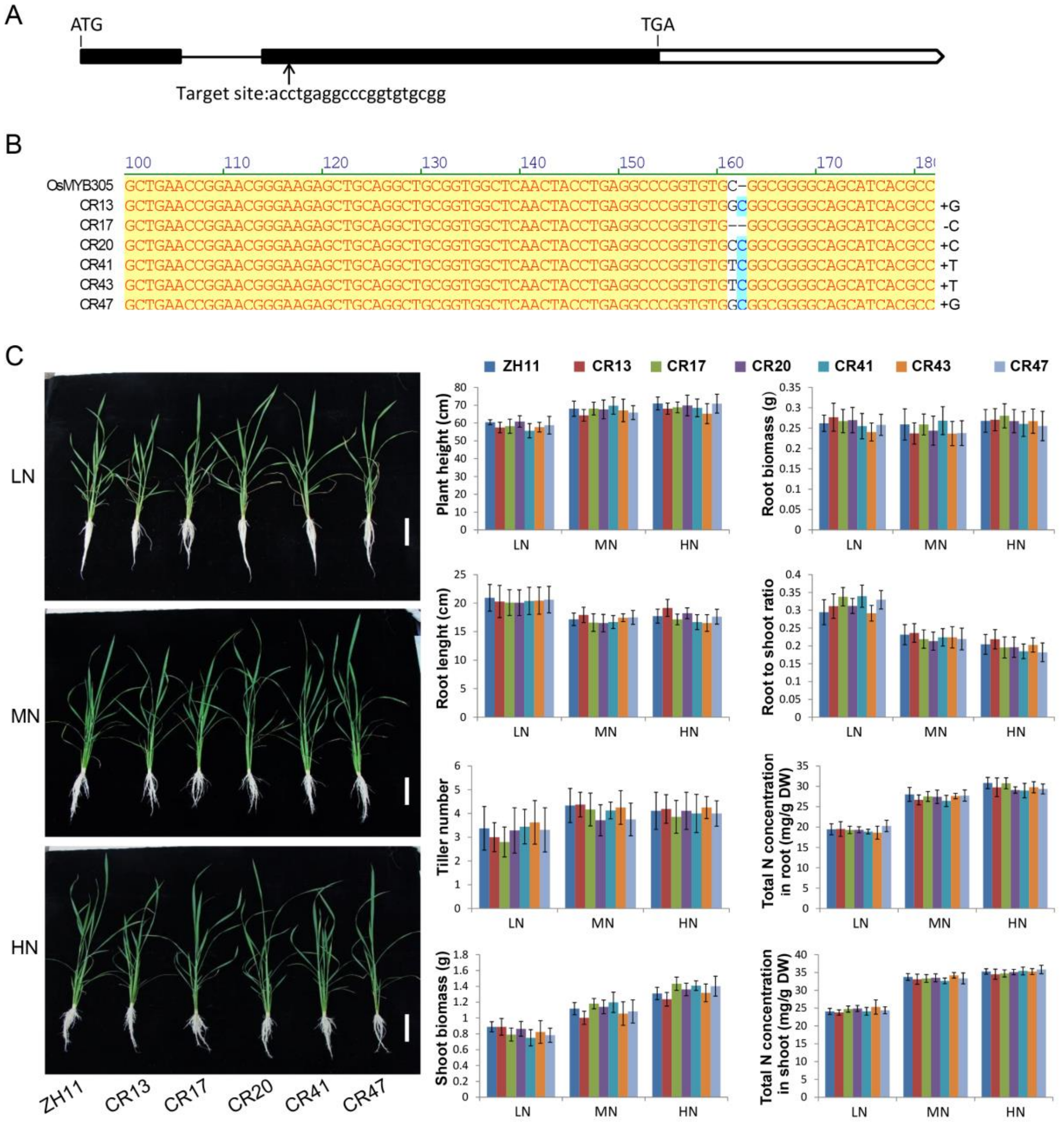

Figure S6. The phenotypes of *OsMYB305* mutants. **(A)** The schematic of the design for *OsMYB305* CRISPR mutants. **(B)** Identification of the *OsMYB305* CRISPR mutants (CR13, CR17, CR20, CR41, CR43, CR47) using DNA sequencing. **(C)** The phenotypes of *OsMYB305* mutant lines grown under various nitrogen supplies and statistics analysis of the performances. Data were means ± SD of 6 biological replicates. Statistical significance was determined using Student's *t*-test.

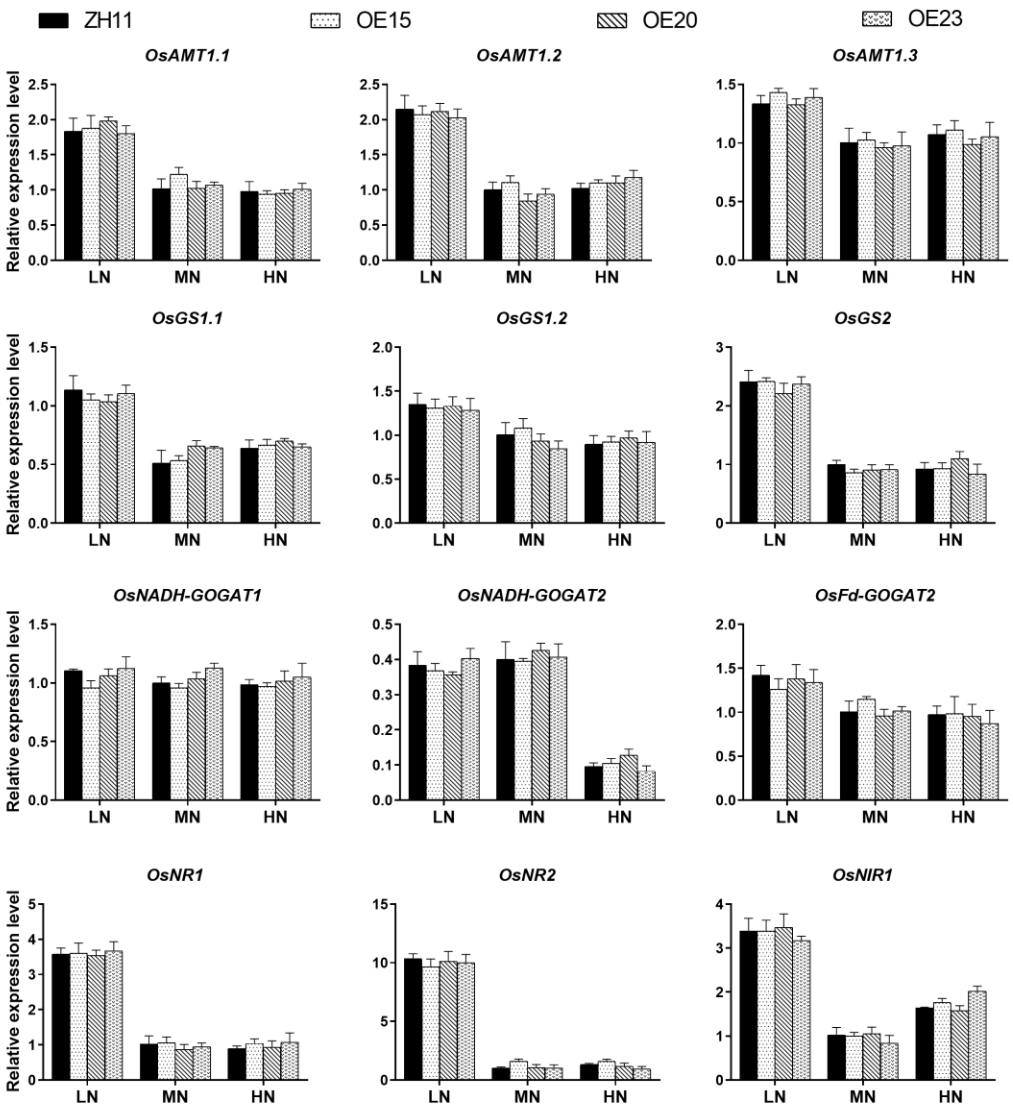

Figure S7. The expression level of genes associated with N uptake and assimilation in the roots of *OsMYB305*-OE lines (OE15, OE20, OE23) and WT (ZH11). Values were means ± SD of 3 biological replications. Statistical significance was determined using Student's *t*-test.

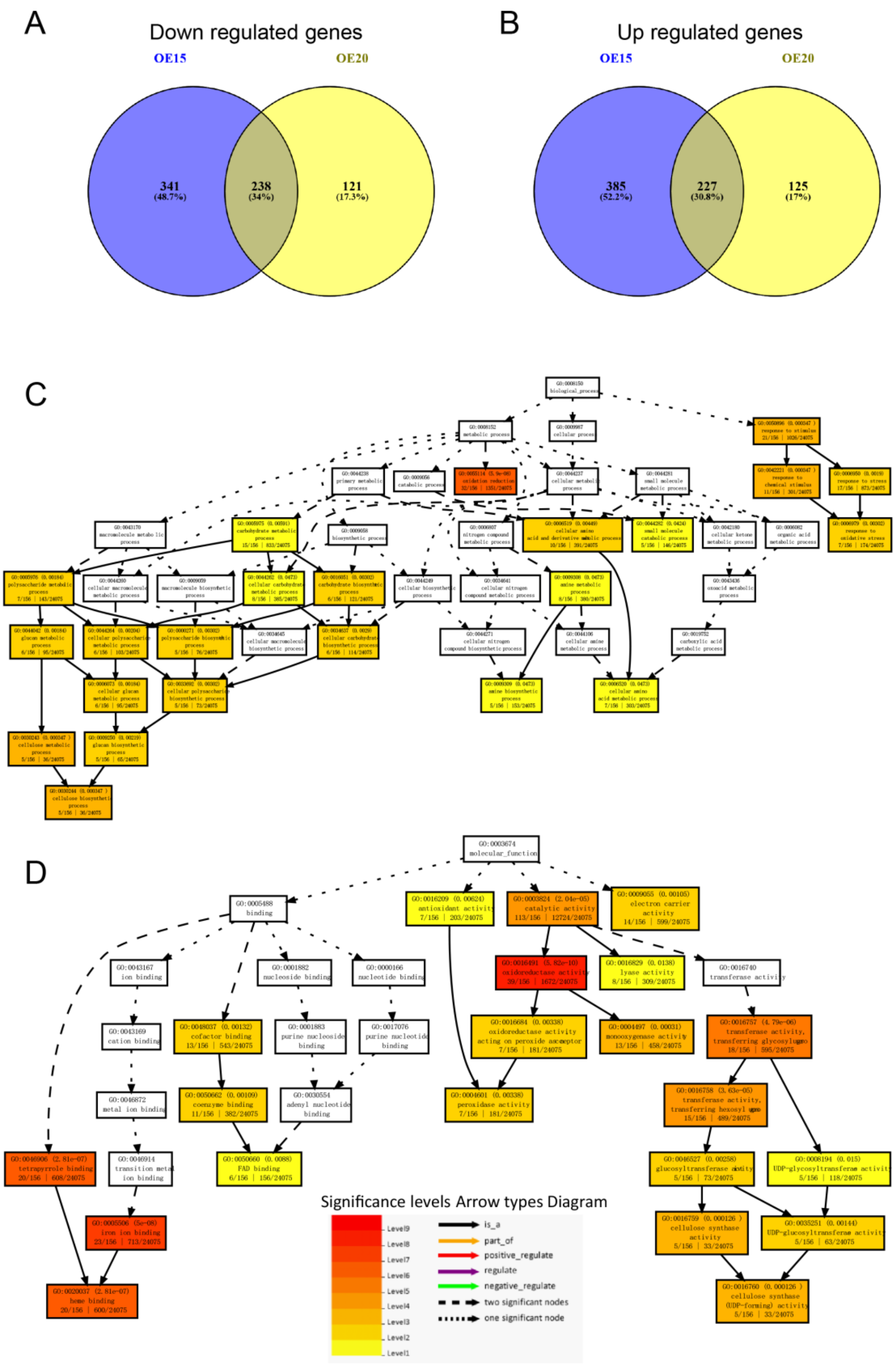

Figure S8. Transcriptome analysis using the roots of *OsMYB305*-OE lines growing under low N condition. **(A, B)** The number of the differential expression genes in the roots of OE15 and OE20. **(C, D)** The GO enrichment analysis of the down-regulated genes. The GO analysis was performed on the website (http://systemsbiology.cau.edu.cn/agriGOv2/). **(C)** The GO terms significantly enriched in "biological process". **(D)** The GO terms significantly enriched in "molecular function".

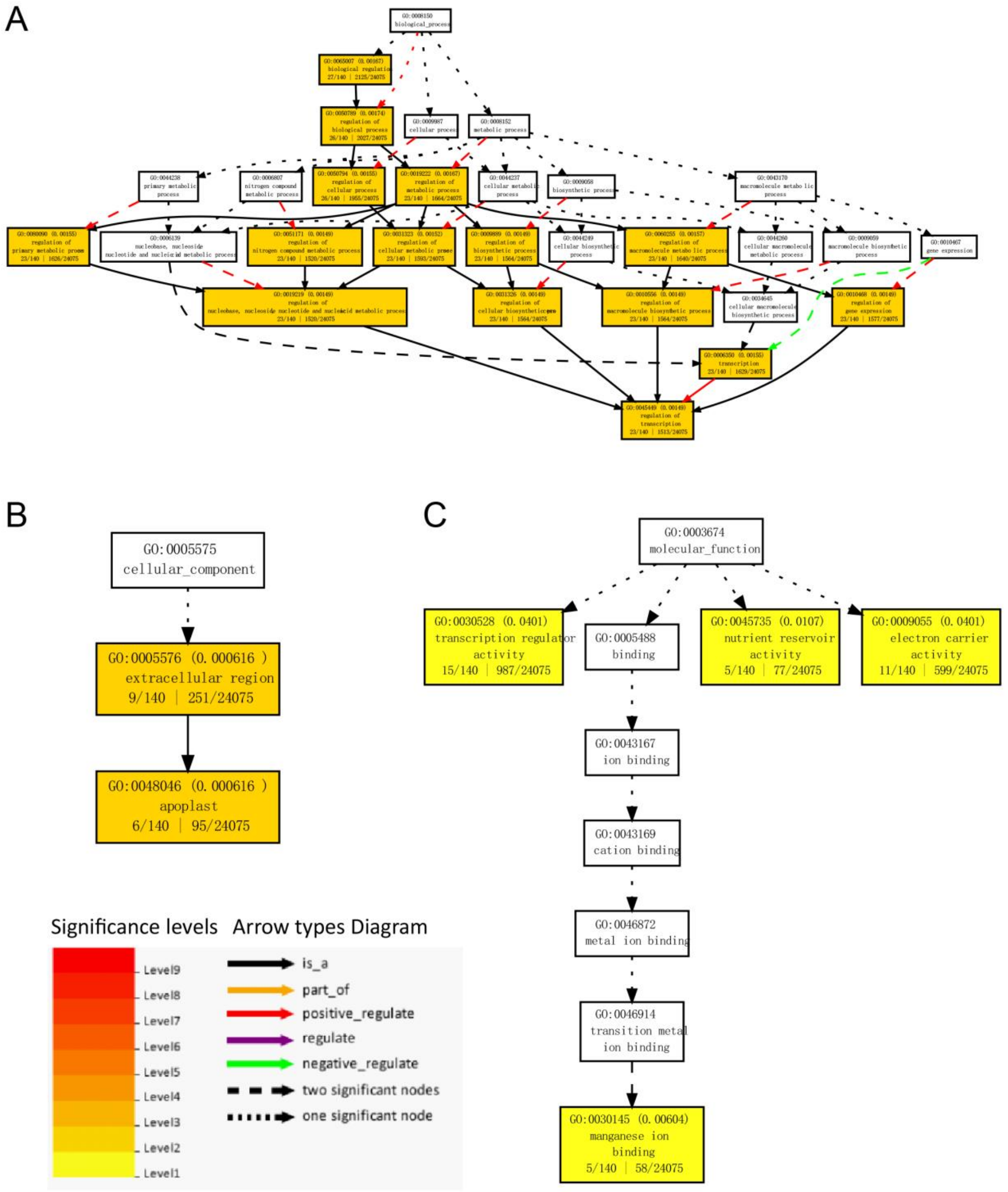

Figure S9. The GO enrichment analysis of the up-regulated genes. **(A)** The GO terms significantly enriched on the "biological process". **(B)** The GO terms significantly enriched on the "cellular component". **(C)** The GO terms significantly enriched on the "molecular function".

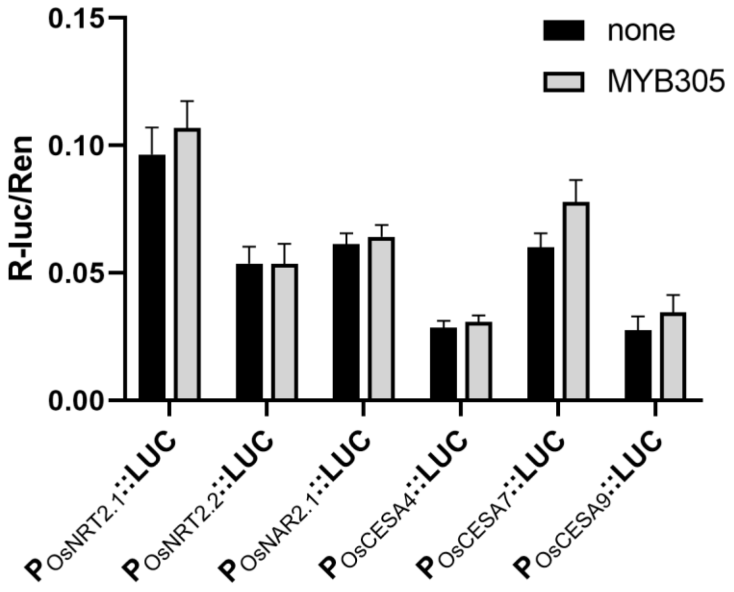

Figure S10. The effects of OsMYB305 on the expression of the DEGs via dual-luciferase reporter assay. The reporters were constructed using the 2 kb fragments of the promoters of the differentially expressed genes. None: empty vector of the effector. The reporter containing Renilla luciferase was used as internal reference. Values were means ± SD of 3 biological replications. Statistical significance was determined using Student's *t*-test.

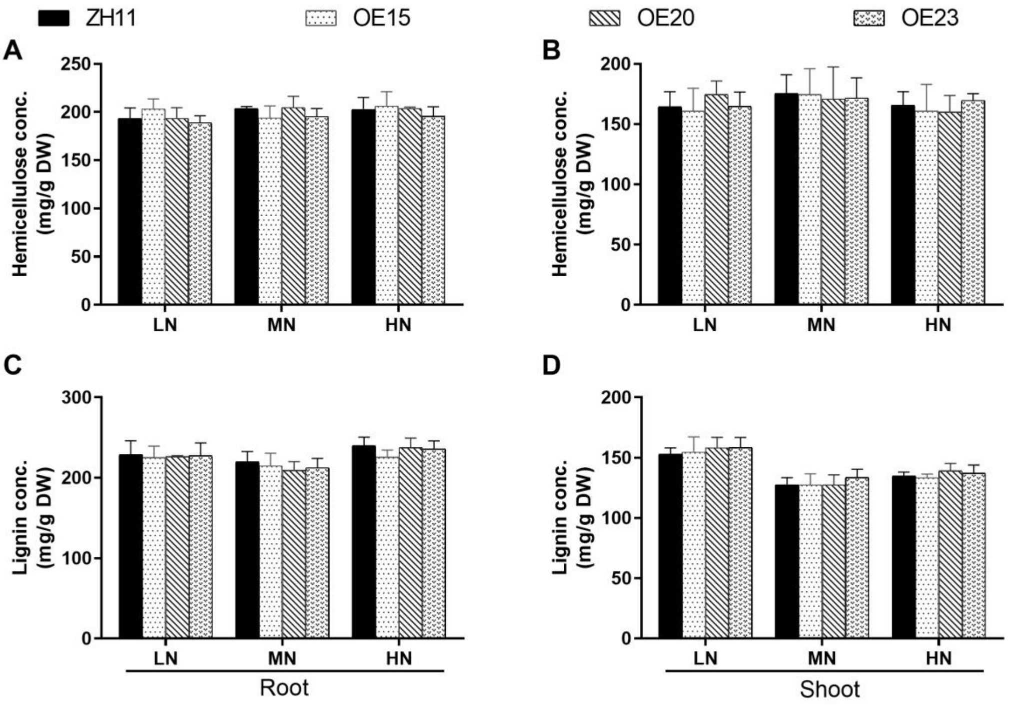

Figure S11. The hemicellulose and lignin concentrations in *OsMYB305*-OE lines (OE15, OE20, OE23) and WT (ZH11). **(A, B)** The hemicellulose concentrations in the roots and shoots. **(C, D)** The Acetyl-bromine lignin concentrations in the roots and shoots. Conc.: concentrations. Values were means ± SD from 3 biological replications. Statistical significance was determined using Student's *t*-test.

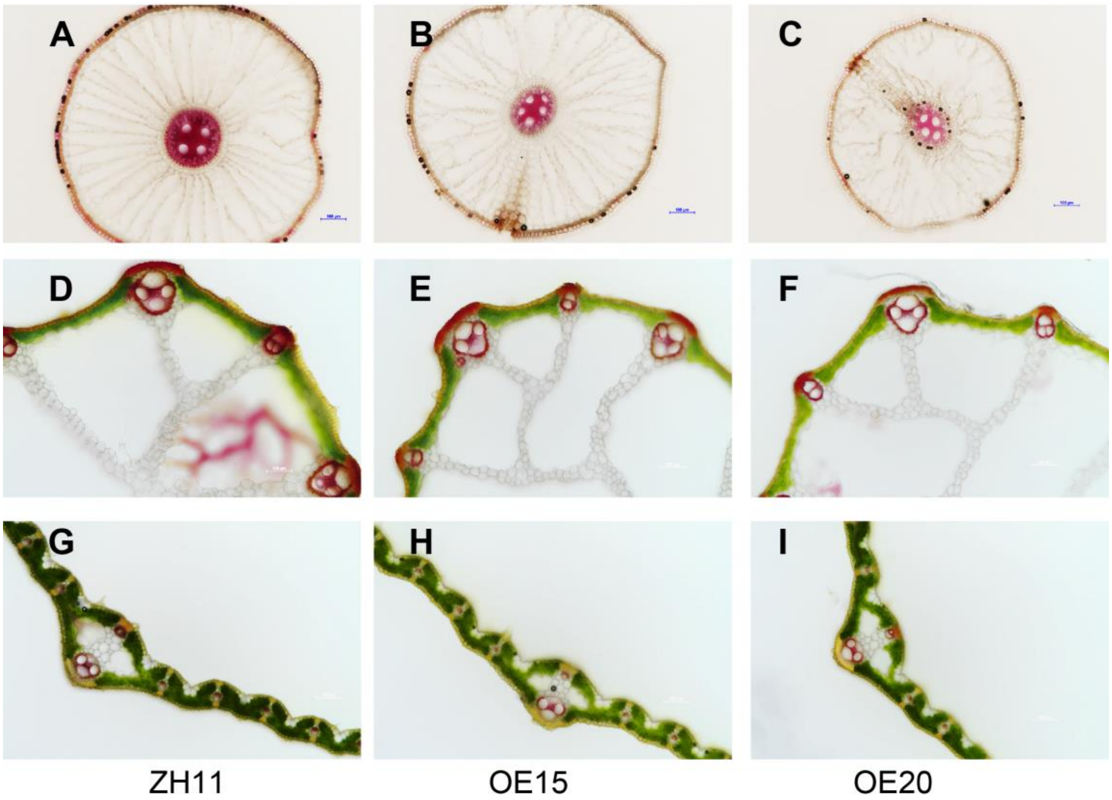

Figure S12. Phloroglucinol dying of the freehand sections from various organs in *OsMYB305*-OE lines (OE15, OE20) and WT (ZH11). **(A-C)** Sections of mature roots. **(D-F)** Sections of leaf sheaths. **(G-I)** Sections of leaf blades.

.

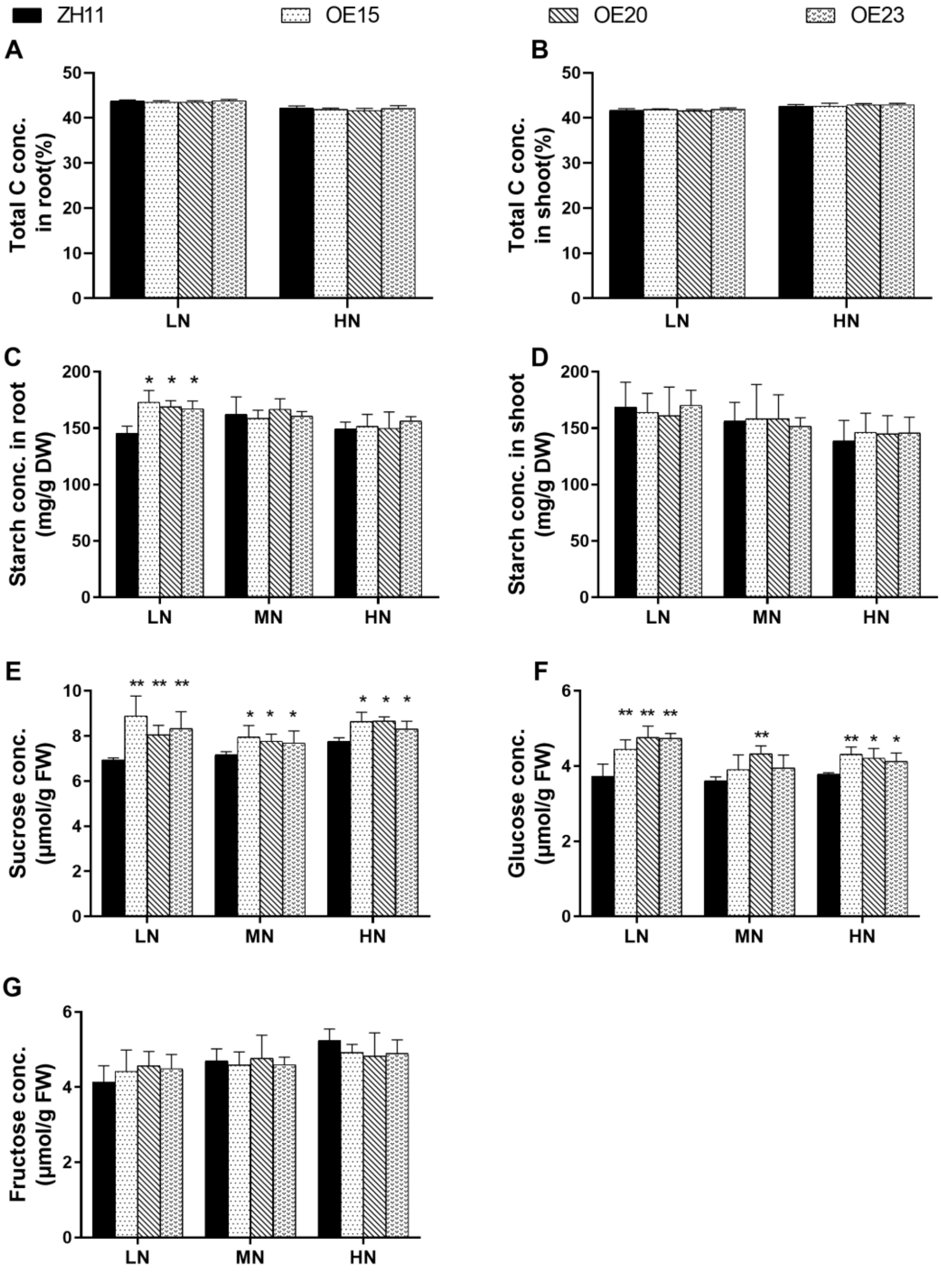

Figure S13. The concentrations of total C and partial C compounds in *OsMYB305*-OE lines (OE15, OE20, OE23) and WT (ZH11). **(A, B)** The total C contents in the roots and shoots. **(C, D)** The starch concentrations in the roots and shoots. **(E-G)** The concentrations of sucrose, glucose and fructose in the roots of *OsMYB305*-OE lines. Conc.: concentrations. Values were means ± SD from 3 biological replications. Statistical significance was determined using Student's *t*-test (* $P < 0.05$, ** $P < 0.01$).

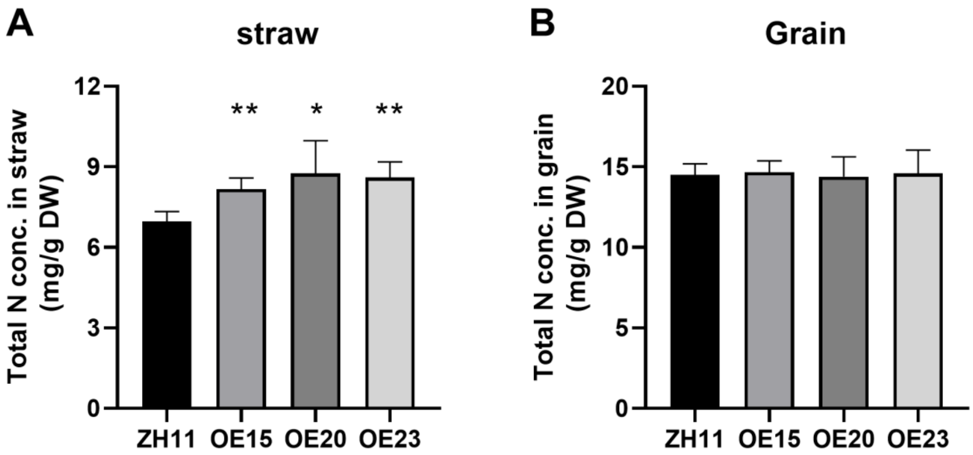

Figure S14. The total N concentrations in straws and grains of *OsMYB305*-OE lines (OE15, OE20, OE23) and WT (ZH11). **(A)** The total N concentrations in the straws. **(B)** The total N concentrations in the grains. The *OsMYB305*-OE lines and WT were planted in the field, supplying with N (urea) 250 kg/hm^2. Values were means ± SD from 10 biological replications. Statistical significance was determined using Student's *t*-test (* *P < 0.05*, ** *P < 0.01*).
